# Variable Copy Number, Intra-Genomic Heterogeneities and Lateral Transfers of the 16S rRNA Gene in *Pseudomonas*

**DOI:** 10.1371/journal.pone.0035647

**Published:** 2012-04-24

**Authors:** Josselin Bodilis, Sandrine Nsigue-Meilo, Ludovic Besaury, Laurent Quillet

**Affiliations:** Laboratoire M2C, Université de Rouen, UMR CNRS 6143, Mont Saint Aignan, France; Texas A&M University, United States of America

## Abstract

Even though the 16S rRNA gene is the most commonly used taxonomic marker in microbial ecology, its poor resolution is still not fully understood at the intra-genus level. In this work, the number of rRNA gene operons, intra-genomic heterogeneities and lateral transfers were investigated at a fine-scale resolution, throughout the *Pseudomonas* genus. In addition to nineteen sequenced *Pseudomonas* strains, we determined the 16S rRNA copy number in four other *Pseudomonas* strains by Southern hybridization and Pulsed-Field Gel Electrophoresis, and studied the intra-genomic heterogeneities by Denaturing Gradient Gel Electrophoresis and sequencing. Although the variable copy number (from four to seven) seems to be correlated with the evolutionary distance, some close strains in the *P. fluorescens* lineage showed a different number of 16S rRNA genes, whereas all the strains in the *P. aeruginosa* lineage displayed the same number of genes (four copies). Further study of the intra-genomic heterogeneities revealed that most of the *Pseudomonas* strains (15 out of 19 strains) had at least two different 16S rRNA alleles. A great difference (5 or 19 nucleotides, essentially grouped near the V1 hypervariable region) was observed only in two sequenced strains. In one of our strains studied (MFY30 strain), we found a difference of 12 nucleotides (grouped in the V3 hypervariable region) between copies of the 16S rRNA gene. Finally, occurrence of partial lateral transfers of the 16S rRNA gene was further investigated in 1803 full-length sequences of *Pseudomonas* available in the databases. Remarkably, we found that the two most variable regions (the V1 and V3 hypervariable regions) had probably been laterally transferred from another evolutionary distant *Pseudomonas* strain for at least 48.3 and 41.6% of the 16S rRNA sequences, respectively. In conclusion, we strongly recommend removing these regions of the 16S rRNA gene during the intra-genus diversity studies.

## Introduction

The 16S rRNA gene is the gold standard for the determination of phylogenetic relationships and environmental diversities because it is universally conserved in all organisms and is thought to be only weakly affected by horizontal gene transfer [1], [2]. The 16S rRNA gene sequence displays an alternating pattern of conserved and hypervariable regions. Since the conserved regions allow the design of universal (as far as possible) primers for PCR amplifications, the hypervariable regions increase the variability, and thus, *a priori*, the resolution of this taxonomic marker.

The rRNA genes of *Bacteria* are usually organized into operons and are cotranscribed in the direction 16S→23S→5S. The copy number of rRNA operons per bacterial genome varies from 1 (*Rickettsia prowazekii* and *Mycoplasma pneumoniae*) to 15 (*Clostridium paradoxum*) [3], [4], [5]. Although the number of rRNA operons is not always invariable in the genome of some species [6], [7], [8], comparison of closely-related organisms reveals that they usually have similar numbers of ribosomal RNA genes [9], [10]. The ability to adapt quickly to changing environmental conditions may provide the selective pressure for the persistence of several rRNA operons in different species of bacteria [11].

Due to gene conversion [12], the multiple copies of 16S rRNA genes of a given bacterial strain are generally identical, with the vast majority of 16S rRNA sequences showing less than 1% of different nucleotides [13], [14]. For a few strains, the 16S rRNA sequences can differ by up to several percentage points between operons [14], [15], [16].

Because only a small fraction of environmental bacterial communities can be cultivated by current techniques [17], bacterial diversity is usually estimated directly from the total DNA of environmental samples by molecular methods (e.g. DGGE –Denaturing Gradient Gel Electrophoresis) and/or sequencing (clone library, massive parallel sequencing). Due to technical limits, only a portion of the 16S rRNA gene, targeting one or two hypervariable regions, is generally amplified and studied [18]. While the 16S rRNA gene is the most universal taxonomic marker, a poor resolution of this marker was observed at the intra-genus level for some genera, e.g. *Vibrio* [19] or *Pseudomonas* [20], [21]. Although the taxonomic power of the 16S rRNA gene has been widely investigated in inter-genus diversity studies [22], [23], the versatile resolution of this molecular marker is still not fully understood at the intra-genus level.

The *Pseudomonas* genus is ubiquitous and includes several species of ecological, economic and medical interest, which suggests a remarkable degree of physiological and genetic adaptability [24]. The exceptional adaptive features of this genus have been explained by numerous intra-genomic recombination events, by lateral transfers of genes encoding niche-specific functions, or by several regulatory genes [24], [25].

As deduced from concatenated housekeeping genes (16S rRNA, *rpoD*, *rpoB* and *gyrB* genes), the hundred species in *Pseudomonas* can be grouped into nine groups (the *P. aeruginosa*, *P. oleovorans*, *P. stutzeri*, *P. fluorescens*, *P. syringae*, *P. lutea*, *P. putida*, *P. anguilliseptica* and *P. straminae* groups) [26]. Moreover, several authors found that these groups split into two major well-supported lineages (whatever the genes used), termed *P. fluorescens* and *P. aeruginosa* lineages [26], [27], [28], [29]. In the 16S rRNA phylogeny, we have previously found an additional splitting of the *P. fluorescens* lineage into two well-supported clusters. The three well-supported clusters in the 16S rRNA phylogeny were termed aeruginosa, putida and fluorescens r-clusters (we term “r”-clusters, the clusters based on “r”RNA gene data) [30]. (i) The aeruginosa r-cluster contains the *P. aeruginosa*, *P. oleovorans*, *P. stutzeri*, *P. anguilliseptica* and *P. straminae* groups; (ii) the putida r-cluster corresponds to the *P. putida* group; (iii) the fluorescens r-cluster contains the *P. fluorescens*, *P. syringae* and *P. lutea* groups. Although the fluorescens r-cluster is the largest group in terms of species number (about 60% of the *Pseudomonas* species described), it contains only 35% of the 19 sequenced *Pseudomonas* strains.

In the *Pseudomonas* genus, the copy number of rRNA operons per bacterial genome varies from 4 (*P. aeruginosa*, PAO1 strain) to 7 (*P. putida*, KT2440 strain) [31], [32]. In the fluorescens r-cluster, five rRNA operons have been described in the Pf5, SBW25, NFM421, B738a, DC3000 and 1448a strains, by genome sequencing [33], [34], [35], [36], and in the F113 strain by Southern hybridization [37]. However, only four rRNA operons have been described in the *P. fluorescens* R2f strain by Southern hybridization [38], and six rRNA operons have been described in the Pf0-1 strain by genome sequencing [34].

In this work, by focusing on the *Pseudomonas* genus, we investigated the limits of the 16S rRNA gene in the study of taxonomic diversity at the intra-genus level. We first investigated the variation of the rRNA gene copy number from 19 sequenced *Pseudomonas* strains and from four additional strains belonging to the fluorescens r-cluster (the R2f, MF0, MFY30 and MFY32 strains). The number of rRNA operons in the four additional *Pseudomonas* sp. strains was investigated by using two different techniques: Southern hybridization and Pulsed-Field Gel Electrophoresis (PFGE) with I-*Ceu*I restriction enzyme. Then, in addition to the sequenced genomes, we used Denaturing Gradient Gel Electrophoresis (DGGE) analysis and sequencing in order to study sequence heterogeneities between the 16S rRNA genes throughout the *Pseudomonas* genus. Finally we investigated the phylogenetic occurrence of the different hypervariable motifs throughout the 16S rRNA sequences of 1803 *Pseudomonas* strains available in the databases.

## Results

### Variable 16S rRNA copy number in *Pseudomonas*

Because restriction enzyme digestion could give misleading results [10], three different probes were used to eliminate the possibility of an over-estimation of the rRNA operon copy number (Figure S1). The number of rRNA operons was determined by Southern hybridization analysis of gel-separated restriction digestions using, alternatively, digoxigenin-dUTP-labeled DNA probes complementary to the 16S 5′end, the 16S 3′end or the 23S 3′end rRNA genes. Experiments were carried out at least three separate times. For each strain, the same fragments hybridized with the three rRNA probes, with the exception of genomic DNA cleaved with *Apa*I because this enzyme has a restriction site in the 16S rRNA genes of *Pseudomonas* (Figure S1). For each strain, the same *Apa*I-cleaved fragments hybridized with the 16S 3′end or the 23S 3′end probes. Consequently, for each strain tested, all the 16S and 23S rRNA genes were organized into at least six (R2f, MF0 and MFY32 strains) or seven (MFY30 strain) operons and were co-transcribed in the direction 16S→23S (Table 1). In order to completely exclude the possibility of an under-estimation of the rRNA operon copy number, we attempted to confirm our results by using a second method.

**Table 1:** Number of chromosomal restriction fragments that hybridize with rRNA probes by Southern analysis^a^.

*Pseudomonas* sp. strain	Restriction enzyme							Probable number of rRNA operons
	*Pst*I	*Cla*I	*Mlu*I	*Apa*I^b^	*Apa*I^c^	*Pst*I+*Cla*I	*Pst*I+*Mlu*I	
MF0	6	6	6	6	6	6	6	6
MFY30	7	7	7	7	7	6	7	7
MFY32	6	6	6	4	4	6	6	6
R2f	5–6	6	5	6	4	6	5–6	6

a All the fragments hybridizing with the three rRNA probes (16S 5′end, 16S 3′end, and 23S 3′end) with the exception of *Apa*I cleaved fragments.

b Fragments hybridizing with the 16S 5′end probe.

c Fragments hybridizing with the 16S 3′end and the 23S 3′end probes.

For each of the four *Pseudomonas* sp. strains (plus *P. putida* KT2440 strain as control), genomic DNA was digested by the intron-encoded endonuclease I-*Ceu*I, which has a restriction site in the 23S rRNA genes, but at no other site so far detected [39], followed by separation of fragments by PFGE (Figure S2). The experiment was carried out at least three separate times. The number of fragments obtained corresponds to the number of rRNA operons (corresponding to the number of 23S rRNA genes) (Table 2). Overall, the PFGE results confirm the number of rRNA operons determined by Southern hybridization and show a genome polymorphism in the fluorescens r-cluster. The genome size of our four *Pseudomonas* sp. strains ranged from 5.68 Mb to at least 6.69 Mb (Table 2). Interestingly, the strain with the smallest genome of the four strains (MFY30) has the greatest number of rRNA operons, although genome sizes should be confirmed by digestion with other restriction enzymes.

**Table 2:** Sizes of I-*Ceu*I fragments of the four *Pseudomonas* sp. strains.

Fragment	Fragment size (kb) obtained from the genomic DNA of:				
	MF0	MFY30	MFY32	R2f	KT2440^b^
A	3,000	2,300	3,300	>4,000	2,800 (2,758)
B	1,300	990	920	780	1,150 (1,223)
C	770	690	800	670	950 (1,047)
D	620	600	650	570	650 (627)
E	310	430	310^a^	360	330 (352)
F	100	360		310	150 (170)
G		310			(5)
Number of rRNA operons	6	7	6	6	6 (7)
Estimated genome size (kb)	6,100	5,680	6,290	>6,690	6,030 (6,182)

a The band is a doublet.

b The numbers in parentheses are the real values deduced from the sequenced genome.

Our PFGE results suggested that all fragments larger than 20 kb had been detected (Figure S2). However, we expected difficulty in observing smaller fragments (i.e. under 20 kb), and in fact, the two rRNA operons organized in tandem in the KT2440 strain could not be detected with this technique, either here (Table 2) or previously [40]. Such a peculiarity in the four other strains was excluded by using a long range PCR inverse with a set of primers hybridizing in reverse with the 5′ end of the 16S rRNA gene, and in the forward direction with the 3′end of the 16S rRNA gene (Figure S3).

**Figure 1:**
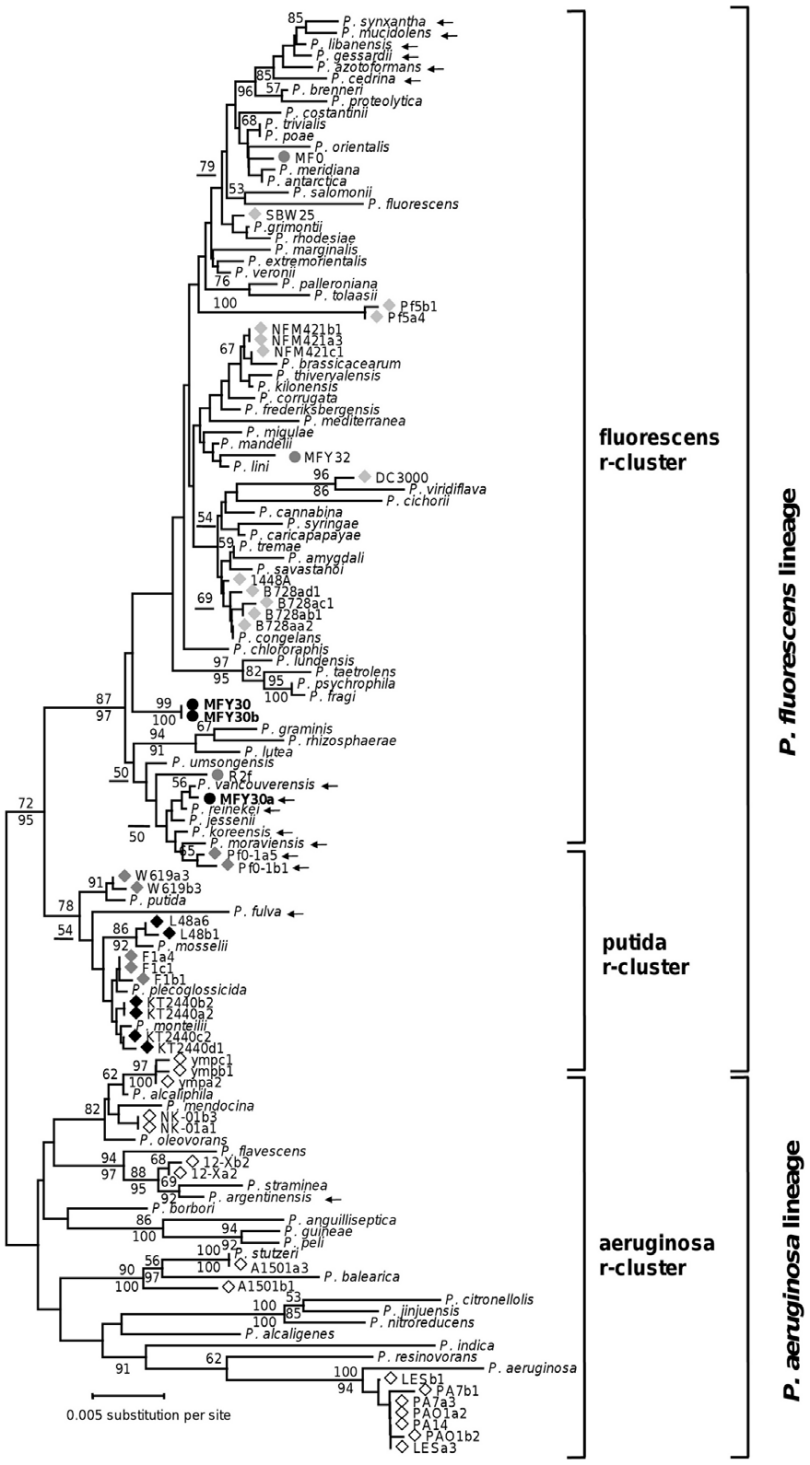
Phylogenetic relationships among 16S ribosomal RNA genes. Sequences from the four strains studied (highlighted by circles), the sequenced strains of *Pseudomonas* (highlighted by diamonds) and 79 *Pseudomonas* type strains were included. The colour of the symbol corresponds to the 16S rRNA copy number in the given strain: white for a strain with 4 copies, light grey for a strain with 5 copies, dark grey for a strain with 6 copies, and black for a strain with 7 copies. The different alleles in the sequenced strains are identified by a letter after the name of the strain, followed by the copy number of this corresponding allele. An arrow marks a sequence with the peculiar motif found in the minor allele a of the MFY30 strain. The unrooted dendrogram was estimated using the Neighbour-Joining algorithm from evolutionary distances computed according to the Jukes and Cantor correction. Numbers on tree branches report bootstrap results from Neighbour-Joining (above branch, 1000 replicates) and Maximum Likelihood (below branch, 100 replicates) analyses for those branches having ≥50% support.

In the *Pseudomonas* genus, there is a very strong correlation between the evolutionary history of the strains and the number of 16S rRNA genes per genome (*P*<0.0001, Spearman test). However, the rate of copy number variation seems very different between the r-clusters: all the strains of the aeruginosa r-cluster have 4 copies of the 16S rRNA gene, those of the putida r-cluster have 6 or 7 copies, while the strains of the fluorescens r-cluster have 5 to 7 copies (Figure 1). More generally, the number of 16S rRNA genes per genome is low and constant in the *P. aeruginosa* lineage, while this number is higher and variable in the *P. fluorescens* lineage (i.e. the fluorescens r-cluster plus the putida r-cluster).

### Intra-genomic heterogeneities of the 16S rRNA gene in *Pseudomonas*

**Figure 2:**
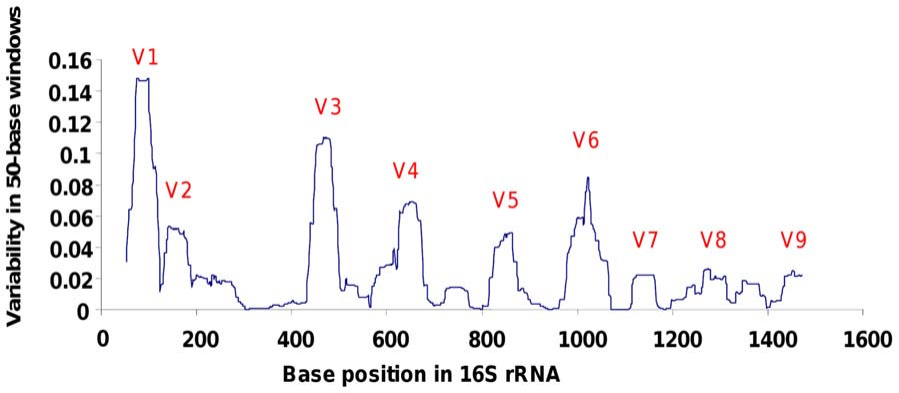
Hypervariable regions within the 16S rRNA gene in *Pseudomonas*. The plotted line reflects fluctuations in variability amongst aligned 16S rRNA gene sequences of 79 *Pseudomonas* type strains. Variability for each base position was generated as follows: one minus the frequency of the most common nucleotide residue. The resulting frequency distribution was then smoothed by taking the mean frequency within a 50-base sliding window, moving 1 base position at a time along the alignment. Peaks correspond to the known V1 to V9 hypervariable regions.

The (V1 to V9) hypervariable regions were defined as the peaks of variability amongst the aligned 16S rRNA gene sequences of *Pseudomonas* type strains (Figure 2) [41]. The positions of these regions were the same when considering the 1803 full-length sequences of *Pseudomonas* available in the databases (data not shown). The three most hypervariable regions (V1, V3 and V6) represent 17.4%, 13.7% and 7.7% of the variability (and so, *a priori*, of the phylogenetic signal) in the full-length 16S rRNA gene, even though the shortest possible regions (only 26, 23 and 26 nucleotides long, respectively) were defined.

Table 3 shows the number of 16S rRNA genes in the sequenced strains, and the number of alleles in the whole sequence and in the three most hypervariable regions (V1, V3 and V6). Overall, most of the *Pseudomonas* strains (15 out of 19 strains) had at least two different 16S rRNA alleles, but a great difference (19 nucleotides) was only observed in one strain (for the *P. stutzeri* A1501 strain). As expected when some mutations or lateral transfers (but not sequencing errors) occurred, all the variable nucleotides were found in the variable regions (i.e. near the hypervariable regions) of the 16S rRNA gene, whatever the strain considered.

**Table 3:** Copy number and heterogeneity of the 16S rRNA gene in the sequenced *Pseudomonas*.

r-cluster	Strain	Genome size (bp)	GC content (%)	16S rRNA copy number	Number of alleles / number of variable nucleotides^a^			
					In the full length	In the three most variable regions^b^		
						V1	V3	V6
**aeruginosa**	LES	6,601,757	66.3	4	2 / 1	1 / 0	1 / 0	1 / 0
	PAO1	6,264,404	66.6	4	2 / 1	1 / 0	1 / 0	1 / 0
	PA7	6,588,339	66.4	4	2 / 2	1 / 0	1 / 0	1 / 0
	PA14	6,537,648	66.3	4	1 / 0	1 / 0	1 / 0	1 / 0
	ymp	5,072,807	64.7	4	3 / 3	2 / 1	1 / 0	1 / 0
	A1501	4,567,418	63.9	4	**2 / 19** c	2 / 5	1 / 0	1 / 0
	NK-01	5,434,353	62.5	4	**2 / 5** c	2 / 5	1 / 0	1 / 0
	12-X	5,048,221	63.6	4	2 / 1	1 / 0	1 / 0	1 / 0
**putida**	L48	5,888,780	64.2	7	2 / 1	1 / 0	1 / 0	1 / 0
	W619	5,774,330	61.4	6	2 / 1	1 / 0	1 / 0	1 / 0
	KT2440	6,181,863	61.5	7	4 / 3	1 / 0	1 / 0	1 / 0
	F1	5,959,964	61.9	6	3 / 2	1 / 0	1 / 0	1 / 0
**fluorescens**	Pf-5	7,074,893	63.3	5	2 / 1	1 / 0	1 / 0	1 / 0
	Pf0-1	6,438,405	60.5	6	2 / 2	1 / 0	1 / 0	1 / 0
	SBW25	6,722,000	60.5	5	1 / 0	1 / 0	1 / 0	1 / 0
	NFM421	6,843,248	60.8	5	3 / 2	1 / 0	1 / 0	1 / 0
	DC3000	6,397,126	58.4	5	1 / 0	1 / 0	1 / 0	1 / 0
	1448A	5,928,787	58	5	1 / 0	1 / 0	1 / 0	1 / 0
	B728a	6,093,698	59.2	5	4 / 3	1 / 0	1 / 0	1 / 0

a Number of different nucleotides between the most divergent alleles.

b Positions 72 to 97 (V1), 453 to 474 (V3) and 993 to 1018 (V6), in the *P. aeruginosa* numbering system.

c Probable lateral transfer.

It is interesting to observe that the variable nucleotides were grouped (at least partially) in the V1 hypervariable region for the two strains having the greatest inter-operonic differences (the *P. stutzeri* A1501 and *P. mendocina* NK-01 strains). For these two strains we found a variation of 5 nucleotides in the V1 hypervariable region. Considering the slow evolution of the 16S rRNA gene and the homogenization of the different 16S rRNA copies by gene conversion, we think that these great intra-genomic differences in the *P. stutzeri* A1501 and *P. mendocina* NK-01 strains probably result from partial lateral transfers. The minor differences between the 16S rRNA genes could be explained in the other strains by a small number of mutations.

As we can see in Figure 1, the intra-genomic heterogeneities observed in the sequenced *Pseudomonas* strains have few phylogenetic consequences. The two alleles of the *P. stutzeri* A1501 strain are, however, closely related to different *Pseudomonas* type strains (*P. stutzeri* and *P. balearica*).

It is important to note that the alignment used to construct the phylogenetic tree shown in Figure 1 was truncated to the same size as the shortest sequence (positions 119 to 1368 in the *P. aeruginosa* numbering system). The V1 hypervariable region was thus excluded. In order to study the phylogenetic consequences of the intra-genomic heterogeneities of the V1 hypervariable region, we carried out a second phylogenetic tree only from the full-length 16S rRNA sequences of *Pseudomonas* type strains and *Pseudomonas* sequenced strains (Figure S6). Compared to the phylogenetic tree in Figure 1, all the partial 16S rRNA sequences were excluded. The topology of both phylogenetic trees (Figure 1 and S6) was almost the same, except that three sequences (corresponding to the *P. peli*, *P. borbori*, and *P. guineae* type strains) were moved from the aeruginosa r-cluster to the fluorescens r-cluster (Figure S6). This incongruence was only found with a Neighbour-Joining analysis, while a Maximum Likelihood analysis, as well as phylogenetic analyses without the V1 region (whatever the phylogenetic method used) gave a phylogenetic tree congruent with the species phylogeny (data not shown). Already, in a recent study, Kampfer and Glaesser have pointed out different tree topologies between Maximum Likelihood and Neighbour-joining trees from the 16S rRNA gene of *Pseudomonas* type strains [42].

Next, we investigated sequence heterogeneities between the 16S rRNA gene copies in our four *Pseudomonas* sp. strains by using the denaturing gradient gel electrophoresis (DGGE). This method was applied by targeting part of the 16S rRNA gene including the V3 hypervariable region (position 341–534). The primers 341f and 534r have been used in many environmental microbiology studies and allow an amplification of the 16S rRNA gene from the whole Bacteria domain [43], [44]. Moreover, since the V3 region is the second most variable region of the 16S rRNA gene in *Pseudomonas* (Figure 2), we expected that many differences in the 16S rRNA genes would be detected by focusing only on this region. For example, although most of the different nucleotides in the A1501 strain are grouped around the V1 region, different alleles should be detected by our DGGE protocol (data not shown).

**Figure 3:**
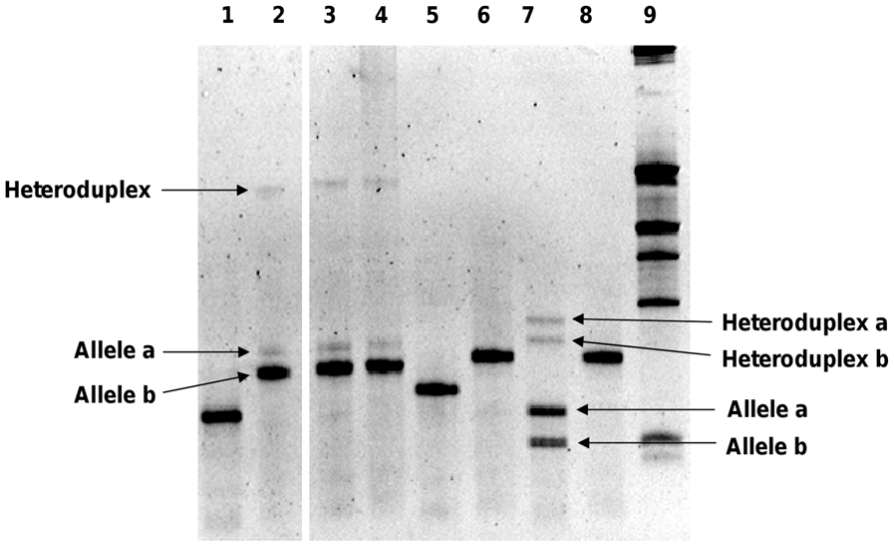
Search for sequence heterogeneity in the V3 region of the 16S rRNA gene by DGGE. Lane 1, MF0 strain; lane 2–4, MFY30 strain; lane 5, MFY32 strain; lane 6, R2f strain; lane 7, KT2440 strain; lane 8, Pf0-1 strain; and lane 9, size ladder (Smart Ladder Small Fragment, Eurogenetec, Belgium).

The sequenced genome of the *P. putida* KT2440 strain also revealed two alleles differing by only one nucleotide in the region amplified by DGGE, near the V3 hypervariable region (data not shown). Five copies of the 16S rRNA gene corresponded to the first allele while the other two corresponded to the second allele. By detecting the two different alleles in the *P. putida* KT2440 strain, our DGGE conditions allowed the maximal resolution (Figure 3).

DGGE analysis based on the V3 region of the MF0, MFY32 and R2f strains showed only one band, revealing that these strains have only one allele of the 16S rRNA gene (Figure 3). In contrast, the MFY30 strain presented three bands with DGGE analysis, highlighting differences between its 16S rRNA genes. Further investigation of the different bands revealed that the highest band was a heteroduplex (noted heteroduplex), while the other two bands (noted alleles a and b) corresponded to two different alleles of the 16S rRNA gene. It is important to note that we carried out this experiment at least three separate times, from extracted genomic DNA or directly from isolated colonies.

To get a better insight, we constructed clone libraries containing the near-full-length 16S rRNA genes of the MFY30 strain. By screening the clones by DGGE, after a nested PCR from a first amplification using universal primers from the vector, we found a proportion of about one copy of the allele a for ten copies of allele b (data not shown). This result is consistent with the difference of intensity observed between the two alleles of MFY30 (Figure 3). It is important to note that we cannot exclude the presence of additional alleles that vary only outside the V3 region. Nevertheless, because previous sequencing of the 16S rRNA gene of MFY30, from PCR amplification without cloning, gave a sequence identical to allele b without ambiguity [30], this allele is certainly the most represented. This means that allele a is a minor one, probably present as a single copy in the MFY30 genome, while allele b is the major allele. For each allele, two 16S rRNA genes, resulting from two independent PCR products, were sequenced and shown to be identical. So, only one sequence of each allele (MFY30a and MFY30b clones) were deposited in gene databases (**GQ411910** and **GQ411911**, respectively) and used for subsequent analysis.

In the whole rRNA gene, a difference of 12 nucleotides was found between the two alleles of MFY30, i.e. 0.8% of the whole 16S rRNA gene and 6.2% considering only the region analyzed by DGGE. Interestingly, these nucleotides were grouped exclusively in the short V3 hypervariable region (23 nucleotides) and presented a similar secondary structure, indicating that allele a was not a pseudogene (Figure S4). This last point was confirmed by RT-PCR followed by a DGGE analysis (data not shown) or by RT-PCR with primers designed specifically for each allele (Figure S5).

As for the *P. stutzeri* A1501 and *P. mendocina* NK-01 strains, we suspect a lateral transfer of the minor allele in MFY30, because the presence of this peculiar motif cannot be explained by an accumulation of point mutations. Lateral transfer may replace an existing copy of the A1501 and NK-01 strains (the closely-related strains have the same 16S rRNA copy number) while insertion of an additional allele could explain the atypical presence of 7 copies of 16S rRNA genes in the *Pseudomonas* sp. MFY30 strain.

Interestingly, the peculiar motif in the minor allele of MFY30 was found in evolutionary close strains but also in more distant strains (Figure 1, Table 4), while it was not present outside the *Pseudomonas* genus. Moreover, in the 16S rRNA gene of *P. jessenii*, a strain close to MFY30 (Figure 1), this motif was identical, except for five ambiguous nucleotides (noted “n”) that probably resulted from the presence of at least two different alleles in the strain. The 16S rRNA heterogeneities in both the *Pseudomonas* sp. MFY30 strain and *P. jessenii* could result from a delay in the 16S rRNA homogenization by gene conversion after a lateral transfer.

**Table 4:** Motifs of the V1, V3 or V6 hypervariable regions found in at least two r-clusters.

Hypervariable region	r-cluster^a^	Proportion of 16S rRNA genes having this motif		Nucleotide sequences
		Among the type strains (%)	Among the set of 1803 sequences (%)	
**V1**	A, F	35,4%	22,1%	tagagagaagcttgcttctcttgag-
	A, F, P	5,1%	14,2%	atgacgggagcttgctccttgattc-
	A, F	0,0%	11,3%	atgaagggagcttgctcctggattc-
	A, F	10,1%	10,5%	agcacgggtacttgtacctggtggcg
	A, F	3,8%	4,6%	atgaagagagcttgctctctgattc-
	A, F	3,8%	3,9%	tagagaggtgcttccacctcttgag-
	F, P	5,1%	2,6%	atgagaagagcttgctcttcgattc-
	A, F	2,5%	0,6%	atgaagggagcttgctcccggattc-
	A, P	0,0%	0,3%	atgatgggagcttgctcctggattc-
	F, P	0,0%	0,2%	atgaagggagcttgctccttgattc-
	A, F	0,0%	0,2%	tagagaggagcttgcttctcttgag-
**V3**	A, F, P	11,4%	28,5%	cagtaagttaataccttgctgtt
	**A, F, P**	**13,9%**	**13,1%**	**ttgtagattaatactctgcaatt** b
	A, F, P	10,1%	11,6%	cattaacctaatacgttagtgtt
	A, F, P	2,5%	6,7%	cagtaagctaataccttgctgtt
	A, F	3,8%	4,3%	cagtaaattaatactttgctgtt
	A, F, P	6,3%	1,8%	cagtaagcgaataccttgctgtt
	F, P	3,8%	1,4%	tacttacctaatacgtgagtatt
	A, F	0,0%	0,6%	cagtaacttaatacgttgctgtt
	A, P	0,0%	0,5%	ctgtaggctaatatcctgcggtt
	A, P	1,3%	0,4%	cagtaagctaatatcttgctgtt
	A, F	0,0%	0,4%	cattaacctaatacgttagtgct
	A, F	0,0%	0,3%	cagtacattaatactgtgctgtt
	A, P	0,0%	0,2%	cagtaagttaataccttgctgtc
	A, F	0,0%	0,1%	cagtaacctaatacgttattgtt
**V6**	A, P	13,9%	27,7%	gcagagaactttccagagatggattg

a The letters correspond to the aeruginosa (A), fluorescens (F) and putida (P) r-clusters.

b This V3 motif was found in the minor allele (allele a) of the 16S rRNA gene in the MFY30 strain.

Finally, we studied the occurrence and the positions of the 612 ambiguous nucleotides in the 1803 full-length sequences of *Pseudomonas* available in the databases. We found that the proportion of ambiguous nucleotides was three times higher in the V1 and V3 hypervariable regions compared to the full-length sequences (*P*<0.001; z test), while this proportion was only twice as high in the V6 hypervariable regions (*P*<0.01; z test). Since the ambiguous nucleotides are not spread throughout the 16S rRNA gene, which would be the case if they resulted from sequencing errors, these positions may reflect higher mutation and/or lateral transfer rates in the V1 and V3 regions.

### Lateral transfers and mosaic structure of the 16S rRNA gene in *Pseudomonas*

In order to further investigate the occurrence of partial lateral transfers of the 16S rRNA gene, we studied the distribution of the three most variable regions in *Pseudomonas* (V1, V3 and V6 regions) in 1803 full-length sequences of *Pseudomonas* available in the databases. We built phylogenetic trees from the 1803 sequences after excluding the V1 hypervariable region because this region led to incongruence with the species phylogeny (see Figure S6). Since the phylogenetic relationships of the *Pseudomonas* type strains were clearly established from several housekeeping genes [20], [21], [26], [27], [28], the 16S rRNA sequences corresponding to the 79 *Pseudomonas* type strains included in the 1803-sequence dataset allowed us to validate the topology and to classify all the sequences in the three r-clusters (Figure S7). Note that, even if the nodes between the three r-clusters were poorly supported by bootstrapping (because of the weak resolution of the 16S rRNA gene and the presence of sequences around the r-cluster boundaries), the topologies of the trees were almost identical, whatever the phylogenetic method used (data not shown). Only three sequences had ambiguous positions (i.e. found in different r-clusters according to the phylogenetic method used) and were clearly identified (Figure S7). These three strains were the AsdM6-4, WAB1930 and LB-F strains (the GenBank access numbers are: FM955873, AM184269 and AB495128, respectively). By contrast, phylogenetic analyses from the 1803 full-length sequences (including the V1 region) gave different trees according to the phylogenetic method (Neighbour-Joining or Maximum Likelihood) used, none of the phylogenetic methods grouping correctly the type strains (data not shown). So, from the reference tree shown in Figure S7, we considered that it was unlikely that the presence of a hypervariable motif in two different r-clusters could be the result of a poor phylogenetic reconstruction. In our analysis, the presence of a hypervariable motif in two r-clusters resulted either from mutations (i.e. an evolutionary convergence), from a partial lateral transfer or from an ancestral character inheritance (vertical transfer).

**Figure 4:**
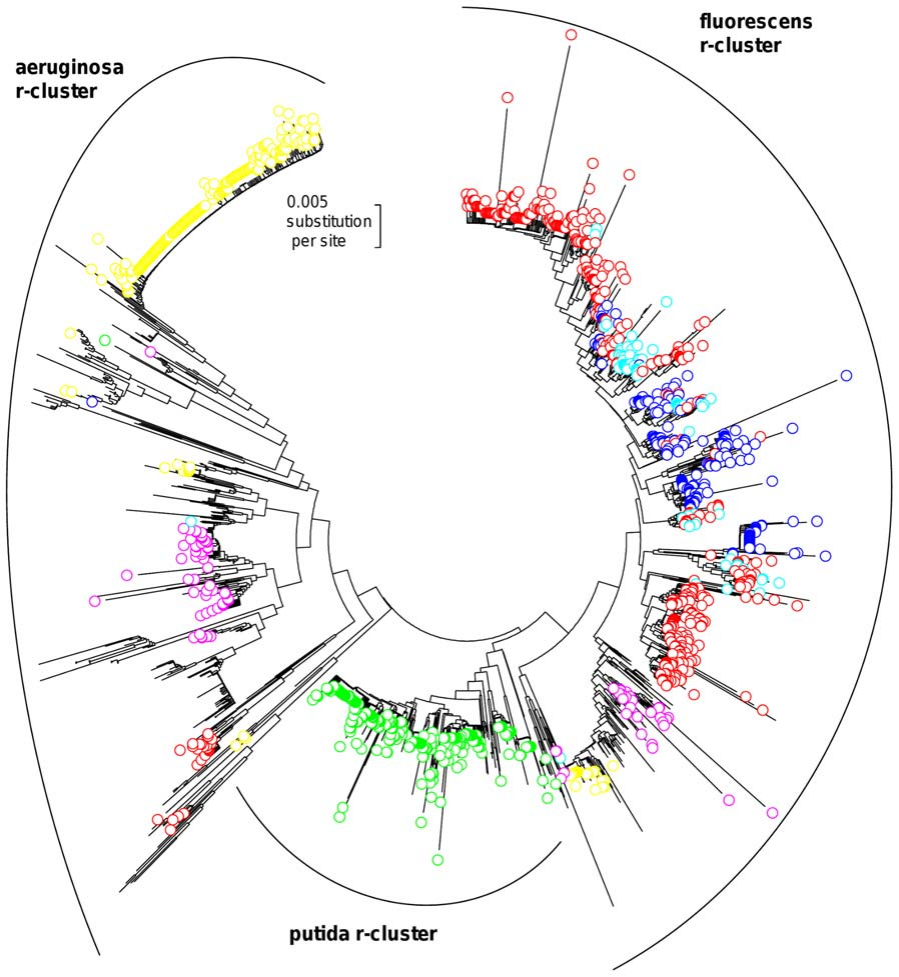
Phylogenetic occurrence of the major V1 hypervariable motifs. The phylogenetic tree was built from 1803 full-length 16S rRNA sequences of *Pseudomonas* available in the databases, after excluding the V1 region (see Figure S7). Each colour corresponds to a V1 motif (present in the original 16S rRNA sequence) found in at least two r-clusters. For the sake of visibility, only the six major V1 motifs (see Table 4) are shown, from the most represented (red circle), in this descending order: red, green, yellow, dark blue, purple, and light blue circles.

The V1 region is the most variable region of the 16S rRNA gene in *Pseudomonas* (Figure 2). Amongst the 164 different motifs in *Pseudomonas*, 110 were found only in one 16S rRNA sequence, while 11 V1 motifs (present in 70.4% of the 1803 sequences) were found in at least two r-clusters (Figure 4, Table 4). Following the most parsimonious hypothesis, that the most represented V1 motif (corresponding to 22.1% of the 1803 sequences) is the ancestral motif in *Pseudomonas*, then at least 48.3% of the 16S rRNA genes in the *Pseudomonas* genus have the same V1 motif as another evolutionary distant *Pseudomonas* strain. These redundancies must result either from an evolutionary convergence or from a partial lateral transfer. Moreover, 13 motifs amongst the 164 different V1 motifs (corresponding to 13.0% of the 1803 sequences) were also found in other bacterial genera (BLAST search).

**Figure 5:**
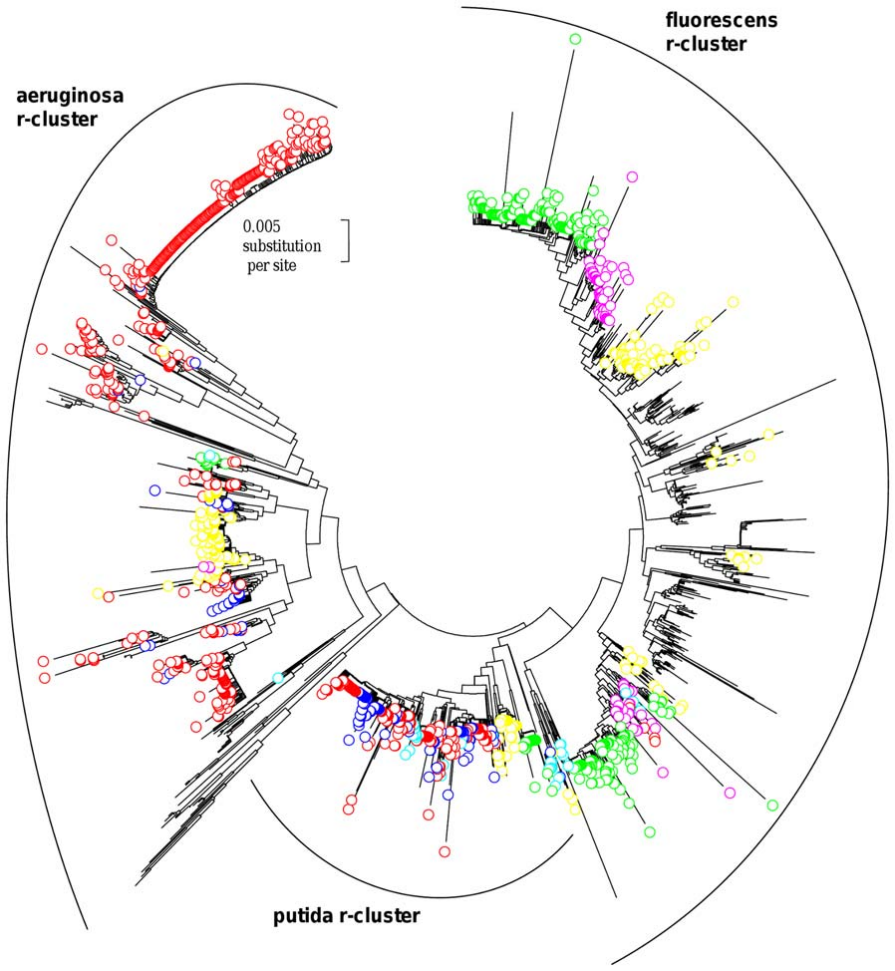
Phylogenetic occurrence of the major V3 hypervariable motifs. The phylogenetic tree was built from 1803 full-length 16S rRNA sequences of *Pseudomonas* available in the databases, after excluding the V1 region (see Figure S7). Each colour corresponds to a V3 motif found in at least two r-clusters. For the sake of visibility, only the six major V3 motifs (see Table 4) are shown, from the most represented (red circle), in this descending order: red, green, yellow, dark blue, purple, and light blue circles. The sequences highlighted by a green circle have the V3 motif found in the minor allele (allele a) of the 16S rRNA gene in the MFY30 strain.

The V3 region is the second most variable region of the 16S rRNA gene in *Pseudomonas* (Figure 2). Amongst the 146 different motifs in *Pseudomonas*, 92 were found only in one sequence, while 14 V3 motifs (present in 70.0% of the 1803 sequences) were found in at least two r-clusters (Figure 5, Table 4). Following the most parsimonious hypothesis, that the most represented V3 motif (corresponding to 28.4% of the 1803 sequences) is the ancestral motif in *Pseudomonas*, then at least 41.6% of the 16S rRNA genes in the *Pseudomonas* genus have the same V3 motif as another evolutionary distant *Pseudomonas* strain. These redundancies must result either from an evolutionary convergence or from a partial lateral transfer. Moreover, only one motif amongst the 146 different V3 motifs (corresponding to 0.1% of the 1803 sequences) was also found in other bacterial genera.

The V6 region is the third most variable region of the 16S rRNA gene in *Pseudomonas* (Figure 2). Amongst the 98 different motifs in *Pseudomonas*, 69 were found only in one sequence, while only one V6 motif (present in 27.7% of the 1803 sequences) were found in at least two r-clusters (Figure S8, Table 4). Following the most parsimonious hypothesis, that the most represented V6 motif (i.e. the only V6 motif present in at least two r-clusters) is the ancestral motif in *Pseudomonas*, we found no clue to an intra-genus lateral transfer (or to an evolutionary convergence) of the V6 motif in the *Pseudomonas* genus. However, 21 motifs amongst the 98 different V6 motifs (corresponding to 48.3% of the 1803 sequences) were also found in other bacterial genera.

Altogether, we found that the great majority (95.2%) of the 16S rRNA genes in *Pseudomonas* presented a mosaic structure because they have the same (V1, V3 and/or V6) motif as another evolutionary distant *Pseudomonas* strain, resulting from a partial lateral transfer, an ancestral character inheritance, or an evolutionary convergence.

## Discussion

### Variable copy number of the 16S rRNA gene

In this study, we found six or seven rRNA operons in four different strains of the fluorescens r-cluster by using two different techniques. Interestingly, we detected six rRNA operons in the R2f strain with a high degree of confidence, whereas Binnerup et al. [38] had previously described only four rRNA operons in this strain, thus highlighting the difficulty of determining the rRNA operon copy number using only one method. It should be noted that real-time PCR could also be an alternative method to determine the gene copy number per genome [7], [45] and, obviously, genome sequencing will continue to provide data about the variation of the copy number of the 16S rRNA gene within species and genera [10].

Our study at a fine-scale resolution has highlighted a variation of the 16S rRNA copy number between closely-related strains, but also a different rate of copy number variability between two close phylogenetic lineages: all the strains of the *P. aeruginosa* lineage have 4 copies of the 16S rRNA gene, while the strains of the *P. fluorescens* lineage (the fluorescens r-cluster plus the putida r-cluster) have 5 to 7 copies.

Because the *P. aeruginosa* lineage is the most variable lineage (in terms of mean divergence and maximal pairwise distance, based on the 16S rRNA sequences; data not shown), a higher variation of the 16S rRNA copy number was expected in this lineage. We propose two hypotheses to explain the unexpected stability of the copy number in the *P. aeruginosa* lineage, compared to the other lineage (or to explain the unexpected variability of copy number in the other lineage). (i) Since it has been suggested that the rRNA number per genome in a bacterial community correlates with the rate of adaptation to a change in the nutrient source [11], a different copy number of rRNA in the *Pseudomonas* genus might reflect a different ecological strategy between species belonging to the different lineages, and more precisely, a more versatile ecological strategy in *P. fluorescens* lineage. (ii) There could be a molecular system (yet to be found) for regulating the number of 16S rRNA genes in the bacteria, but that is absent or modified in the strains of the *P. fluorescens* lineage (the fluorescens and putida r-clusters).

### Intra-genomic heterogeneities

Intra-genomic heterogeneities have been reported for about half of the sequenced bacterial species having at least two 16S rRNA genes [14]. For a few species, the intra-genomic heterogeneities were higher than several percentage points [15], [16], [46], while only low micro-heterogeneities (i.e. lower than one percent) were found for the great majority of the other species. In our study, we found that these intra-genomic micro-heterogeneities in *Pseudomonas* were essentially localised in the hypervariable regions of the 16S rRNA gene. Interestingly, this localisation of intra-genomic micro-heterogeneities is probably common to the whole Bacteria domain [14], [47]. Since it is quite evident that great intra-genomic diversities probably result from lateral transfers, it is difficult to identify the origin of the low micro-heterogeneities (lateral transfer or evolutionary divergence). Because the motifs suspected to be laterally transferred can be found in several phylogenetic lineages, some authors have proposed that these micro-heterogeneities result from lateral transfers [48], [49], [50]. In agreement with these authors, we argue that the variability observed between alleles of the 16S rRNA gene in the MFY30, A1501 and NK-01 strains probably results from a lateral transfer in the *Pseudomonas* genus.

Moreover, we have found that the proportion of these intra-genomic heterogeneities resulting from a lateral transfer seems to be correlated with the divergence of the different regions concerned (V1>V3>V6 for the *Pseudomonas* genus). For the *Pseudomonas* genus, we have found that 10.5% (2 strains out of 19), 4.3% (1 strain out of 23) and 0% (0 out of 19) of the *Pseudomonas* strains present intra-genomic heterogeneities resulting from lateral transfers in the V1, V3 and V6 hypervariable regions, respectively. Even though the number of strains used for these last estimations is small (i.e. the sequenced strains plus our four studied strains), this tendency was the same when the positions of the ambiguous nucleotides (noted “n”) in the 1803 full-length sequences of *Pseudomonas* available in the databases were studied, i.e. the V1 hypervariable region had the highest number of ambiguous nucleotides, followed by the V3 hypervariable region, and then the V6 hypervariable region.

More generally, these intra-genomic heterogeneities could be a transitory state after a lateral transfer event and before a gene conversion. For each bacterial genus, the proportion of heterogeneities could be linked to the probability of lateral transfers, to the accuracy of the gene conversion and, as was previously observed in several bacterial genera, to the fitness associated with the different 16S rRNA alleles [51], [52]. In our study, we did not find a link between the number of 16S rRNA genes and the proportion of intra-genomic heterogeneities, but our sample was probably too small to conclude definitively.

The accuracy of studies using environmental DNA (DGGE, clone libraries, massive parallel sequencing) could be influenced by these intra-genomic micro-heterogeneities and thus the real diversity could be over-estimated. Due to the presence of heteroduplexes (during DGGE analyses) and chimerical structures (during amplification for sequencing), this bias could be even higher.

### Lateral transfers and mosaic structure of the 16S rRNA gene

In our study, we have found that the 16S rRNA genes in *Pseudomonas* present an unexpected mosaic structure, with 48.3% and 41.6% of the 16S rRNA genes in the *Pseudomonas* genus having the same V1 and V3 motif as another evolutionary distant *Pseudomonas* strain. This results either from evolutionary convergence or from partial lateral transfer. It is important to note that we used a conservative strategy by considering that the most representative motif is the ancestral motif, and by excluding from our study the intra-cluster redundancies. Consequently, we think that more 16S rRNA genes could be impacted by lateral transfer and/or evolutionary convergence.

The difficult question was whether the occurrence of the same motif in evolutionary distant strains (in different r-clusters or in different bacterial genera) resulted from lateral transfer or from evolutionary convergence. Our reasoning was as follows. First, the more variable the nucleic region, the weaker the evolutionary constraints (i.e. interactions with ribosomal proteins and/or proximity to the active sites) [53], and the greater the probability of lateral transfers [2]. Conversely, the more constant the nucleic region, the lower the number of possible motifs (with the same fitness), the shorter the evolutionary pathway between two motifs, and the greater the probability of obtaining the same motif by chance (i.e. by evolutionary convergence). Secondly, the probability of lateral transfers should be higher between closely-related strains (e.g. in a same bacterial genus) because of a complex coevolution between ribosomal RNAs and proteins [2]. Thirdly, we think that too many independent mutations, in too short an evolutionary time, would be necessary to explain the intra-genomic heterogeneities observed in the A1501, NK-01, and MFY30 strains.

So, according to the occurrence of the different V1, V3 and V6 motifs throughout the *Pseudomonas* genus and throughout the Bacteria domain, we argue that the occurrence of the same V1 or V3 motif in different r-clusters probably results from a lateral transfer, while the occurrence of the same V6 motif outside the *Pseudomonas* genus is most likely to result from an evolutionary convergence. Finally, we cannot decide between lateral transfer and evolutionary convergence concerning the occurrence of the same V1 motif outside the *Pseudomonas* genus.

Even though lateral transfers have been already described in *Pseudomonas* for many niche-specific genes [24] and even for some housekeeping genes [54], it is quite surprising that the occurrence of lateral transfers of the 16S rRNA gene was so high. To explain this phenomenon, we suggest that the genomic integration of a xenologous gene (or a part of a xenologous gene) by crossing over would be facilitated for the 16S rRNA gene because there are several copies of the gene and an alternating pattern of identical and variable regions [55]. Because we found no selective advantage for the transfers, we think, by default, that these lateral transfers of the 16S rRNA gene are evolutionary neutral, as a collateral effect of the gene conversion acting to homogenize the different copies of the 16S rRNA gene. However, since some studies had reported a link between intra-genomic 16S rRNA gene divergence and adaptation to different temperatures [51] it would be interesting to further investigate the fitness associated with these lateral transfers.

Moreover, previous studies found few redundancies of the hypervariable regions between strains belonging to different genera [19], and comparable phylogenetic information (in inter-genus diversity studies) between the V1–V3 region and the full-length 16S rRNA gene [23]. Consequently, the structural constraints of the 16S rRNA gene [2] seem to limit these transfers only between closely-related strains (i.e. in the same bacterial genus). As described previously [20], [21], the taxonomic resolution of the 16S rRNA gene does not allow a robust species identification in the *Pseudomonas* genus, even by considering the full-length sequences. However, only a partial 16S rRNA sequence of *Pseudomonas* is often enough to identify unambiguously a strain or an environmental sequence at the genus level (e.g. by using the Ribosomal Database Project [http://rdp.cme.msu.edu/index.jsp]). The paradox highlighted in our study is that the regions the most impacted by intra-genus lateral transfers (the V1 and V3 hypervariable regions) are probably the best ones (even for the *Pseudomonas* genus) to be targeted for an unambiguous identification at the genus level, because these regions are the most variable and few, if any, lateral transfers (or evolutionary convergences) have been detected between the *Pseudomonas* and other genera.

The detection of lateral transfers only between closely-related strains is coherent with the intra-genomic micro-heterogeneities observed in *Pseudomonas* (this study) and in other bacterial genera [14]. Since we suggest that these intra-genomic micro-heterogeneities are only a transitory state after a lateral transfer and before a gene conversion (i.e. not all the ancient lateral transfers could be detected by studying only the intra-genomic heterogeneities), probably most of the bacterial genera could be strongly impacted by partial intra-genus lateral transfers of the 16S rRNA gene.

### Recommendation for phylogenetic analyses using the 16S rRNA gene

Since, the V1 and V3 regions that represent an important part of the 16S rRNA variability (17.4% and 13.7%, respectively) are strongly impacted by lateral transfers, we recommend removing these regions of the sequence alignments during studies at a fine taxonomical level, when the full-length sequence is used and especially when only a part of the 16S rRNA gene is used (e.g. in massive parallel sequencing). These recommendations partially overlap those from previous studies [28], [56], [57]. In these studies, the authors have recommended removing some hypervariable regions, including V1 and V3 regions, because they are not homologous [57] or they have a variable secondary structure [28], [56].

## Materials and Methods

### Bacterial strains

The *Pseudomonas* strains used in this study were obtained from different sources: the MF0 strain was isolated from raw milk [58], whilst the MFY30 and MFY32 strains were isolated from bulk soil [30]. The R2f strain was isolated from the rhizosphere of wheat [59] and was provided by S. Binnerup. The *P. putida* KT2440 strain, used as a control of known copy number, was isolated from soil [32] and was provided by J. L. Ramos. The API 20 NE test (BioMérieux S.A.) was used to identify the four strains (MF0, MFY30, MFY32 and R2f) as members of the *P. fluorescens* species. Additionally, the 16S rRNA gene was used to further investigate the species affiliation as described in section “Phylogenetic analysis”. The 16S rRNA phylogeny confirms that these strains belong to the fluorescens r-cluster (Figure 1). However, these strains are not close to the *P. fluorescens* type strain in the phylogenetic tree. We thus described them as *Pseudomonas* sp. and not as *Pseudomonas fluorescens*.

### Southern hybridization analysis

Genomic DNA of each *Pseudomonas* sp. strain was extracted by using the Easy-DNA kit (Invitrogen) according to the manufacturer's instructions. About five µg of DNA were digested overnight at 37°C with 80 U of four different restriction enzymes (*Pst*I, *Cla*I, *Mlu*I and *Apa*I) separately or in a double digestion (*Pst*I+*Cla*I or *Pst*I+*Mlu*I). While *Apa*I cuts the 16S rRNA gene of *Pseudomonas*, *Pst*I, *Cla*I and *Mlu*I have no cleavage site in this gene so far detected. The enzymes were provided by Roche. Digested fragments were separated by electrophoresis [0.7% (w/v) agarose gel], transferred by Southern blotting onto Hybond-N+ membrane (Amersham Biosciences) and hybridized to PCR-generated, digoxigenin-labelled probes overnight at 68°C. Revelation was carried out either by colorimetric detection (NBT and BCIP) or by chemiluminescent detection (CDP star). All products for the detection were provided by Roche. The probes 16S 5′ end, 16S 3′ end and 23S 3′ end were synthesized by PCR using DNA extracted from the four *Pseudomonas* sp. strains. They correspond to sequences that are complementary to positions 63–535 and 1069–1522 of the16S rRNA gene and 1910–2764 of the 23S rRNA gene, respectively (in the *E. coli* numbering system).

### PFGE with I-*Ceu*I digestion

Intact DNA was prepared by embedding whole cells in agarose blocks as described previously by [60]. Genomic DNA (0.2 µg or 1 µg) was digested by 5 units of the intron-encoded endonuclease I-*Ceu*I (New England Biolabs), which has a restriction site in the 23S rRNA genes, but no other site in the genome so far detected [39]. Digestion was carried out overnight at 4°C followed by 3 h at 37°C. The resulting fragments were separated on a CHEF electrophoresis (Pharmacia-LKB Gene Navigator).

### Identification of tandem repeat

Genomic DNAs were amplified by PCR using 28r (CTG AGC CAG GAT CAA ACT CT) and 1521f (TGC GGC TGG ATC ACC TCC TT) which correspond to the inverse-complementary of the universal 16S rRNA primers 8f and 1541r (Figure S1). The PCR mixtures (25 µL) contained 0.4 µM of each primer, 300 µM of each deoxynucleoside triphosphate, Crimson LongAmp *Taq* PCR buffer (New England Biolabs), 5 U of the Crimson LongAmp *Taq* DNA polymerase (New England Biolabs) and 400 ng of genomic DNA. PCR was conducted using a GeneAmp PCR System 9700 (Perkin-Elmer Corporation, USA) programmed to the following conditions: 30 seconds denaturation at 95°C, followed by 25 cycles of 10 s at 95°C (denaturation), 30 s at 65°C (annealing) and 16 min at 65°C (extension), with a final 10-min extension step at 65°C after cycling was completed. Amplification was checked by agar gel electrophoresis (1% w/v in 0.5× TAE buffer at 100 V) and ethidium bromide staining.

To test this protocol, we carried out an additional amplification in the same conditions except that the 1521f primer was replaced by the KT20kb primer (TAG GCA ACC CGT TCG ATA CT), a primer designed especially to obtain a PCR product of about 20 kb from the KT2440 strain (in combination with the 28r primer).

### Denaturing gradient gel electrophoresis (DGGE) analysis

The variable V3 region (*Escherichia coli* positions 341–534) of the 16S rRNA genes was analysed by denaturing gradient gel electrophoresis (DGGE) using a previously described protocol [43]. PCR products were analysed by DGGE using the Bio-Rad D Code system, on gels consisting of 8% (w/v) polyacrylamide (acrylamide∶ bisacrylamide, 37∶1) and a denaturing gradient from 40% to 60% [100% denaturant is 7 M electrophoresis grade urea (Sigma) and 40% v/v formamide (Fluka)], in 1× TAE (40 mM Tris-acetate, 1 mM di-sodium-EDTA, pH 8.0), at a constant 80 V and 60°C for 5 h. Gels were visualized under UV at 302 nm using the Alphamager System EU after staining in 500 mL of 0.25 µg. mL^−1^ BET in 1× TAE for 15 min.

### Clone library from the 16S rRNA genes of the MFY30 strain

A DNA clone library was constructed from the MFY30 strain. Near-full-length 16S rRNA genes were amplified using universal primers 8f (AGA GTT TGA TCC TGG CTC AG) and 1541r (AAG GAG GTG ATC CAG CCG CA) and high fidelity *Phusion* hot start DNA polymerase (Finnzymes). Modified PCR products (after adding adenines) were cloned into the pGEMT plasmid using a TA cloning kit (Invitrogen) according to the manufacturer's instructions. The sequence of each copy of the 16S rRNA gene (MFY30a and MFY30b clones) were deposited in databases [GenBank: **GQ411910** and **GQ411911**, respectively].

### Expression of the different 16S rRNA genes in the MFY30 strain

An LB culture (50 mL, DO580nm = 1) of the MFY30 strain was centrifuged at 5000 rpm for 10 min. RNA was extracted from the pellet by using the Fast RNA Pro Soil-Direct Kit (MP Biochemicals) in combination with the FastPrep FP120 bead beating system (Bio-101 Inc., Ca, USA), according to the manufacturer's instructions. DNA was removed from 16 µL of the total nucleic acid extraction by DNase digestion, using deoxyribonuclease I Amplification Grade kit (Sigma). A control PCR was performed to ensure complete removal of DNA, using primers and PCR conditions as detailed below.

cDNA was produced by reverse transcription of RNA using Enhanced Avian HS RT-PCR Kit (Sigma) as recommended by the manufacturer and by using the reverse primer described in section 2.5. One microlitre of reverse transcription product was subsequently used in the PCR reactions.

Expression of the two different copies (alleles a and b) was evaluated either by DGGE or by PCR with primers specific to each 16S rRNA gene. The sets of primers MFY30f (TCA GAT GAG CCT AGG TCG) / MFY30ar (TGC AGA GTA TTA ATC TAC AAC C) and MFY30f / MFY30br (ACT AAC GTA TTA GGT TAA TGC) led to the amplification of a fragment of the alleles a and b, respectively. PCR conditions were the same for both the primer sets: 3 min at 95°C, 30 cycles at 94°C for 30 s, at 59°C for 30 s and at 72°C for 30 s, with a final elongation for 10 min at 72°C. Amplification of the target rRNA gene was confirmed by agar gel electrophoresis (2% w/v in 0.5× TAE buffer at 100 V) and ethidium bromide staining.

### Phylogenetic analysis

The 16S rRNA gene sequences of the sequenced *Pseudomonas* strains were retrieved from the *Pseudomonas* Genome Database [http://v2.pseudomonas.com] and the Sanger Institute [http://www.sanger.ac.uk/Projects]. The 1803 full-length 16S rRNA sequences, including the 16S rRNA gene sequences of the 79 type strains used as reference for phylogenetic analyses, were retrieved from the Ribosomal Database Project [http://rdp.cme.msu.edu/index.jsp].

To obtain the set of 1803 full-length 16S rRNA sequences used in our study, we first searched all the 16S rRNA genes exclusively sequenced from isolated *Pseudomonas* strains (in order to avoid chimerical sequences). From the 6468 near-full-length sequences (≥1200 bases) of good quality (may 2011, RDP release 10, update 27) in *Pseudomonas*, we selected the 1803 sequences that hybridized *in silico* with the 27f and 1492r universal primers.

The 16S rRNA gene sequences of the four strains studied were obtained with amplification directly from bacterial suspensions, and then double–strand sequenced by Cogenics (Meylan, France). The 16S rRNA gene sequences of the MF0, MFY30 and MFY32 strains were determined in a previous study [30] while the 16S rRNA gene sequence of the R2f strain was obtained in the present study [GenBank: **GQ411912**].

For the different phylogenetic studies, the 16S rRNA sequences were aligned using MUSCLE [61], with default parameters. The alignments were truncated to the same size as the shortest sequence, i.e. positions 119 to 1368 (in the *P. aeruginosa* numbering system) for the phylogenetic tree shown in Figure 1, and positions 27 to 1492 for the other phylogenetic trees.

Finally, the Neighbour-Joining trees were estimated using the Jukes and Cantor correction with MEGA v5.0 [62]. The degree of statistical support for the branches was determined with 1000 bootstrap replicates. The Maximum Likelihood trees were estimated using the rapid bootstrap option (100 replicates) and the CAT model of rate heterogeneity, with RAxML version 7.2.6 [63].

## Supporting Information

Figure S1 **Southern blot analysis of rRNA operons in four** ***Pseudomonas*** **sp. strains.** (**A**) Genomic DNAs were cleaved using restriction enzymes and hybridized with the 23S 3′end probe as described in the text. Lane 1, MF0 strain cleaved with *Pst*I+*Mlu*I; lane 2, MFY30 cleaved with *Apa*I; lane 3, MFY32 cleaved with *Pst*I; lane 4, R2f cleaved with *Cla*I. (**B**) Schematic positions of rRNA probes.
(PDF)

Figure S2 **PFGE analysis of rRNA operons in the four** ***Pseudomonas*** **sp. strains.** (**A**) Running conditions: 100 V for 40 h; pulse times, 80 s for 18 h, 100 s for 10 h, and 120 s for 12 h. Lane 1 and 2, DNA of MFY32 strain (0.2 µg and 1 µg, respectively); lane 3 and 4, DNA of R2f strain (0.2 µg and 1 µg, respectively); lane 5 and 6, DNA of MF0 strain (0.2 µg and 1 µg, respectively); lane 7, lambda DNA concatemers (Biorad, USA); Lane 8 and 9, DNA of MFY30 strain (0.2 µg and 1 µg, respectively); lane 10 and 11, DNA of KT2440 strain (0.2 µg and 1 µg, respectively). (**B**) Running conditions: 80 V for 70 h; linearly ramped pulse from 400 s to 800 s. Lane 1 and 2, DNA of MF0 strain (0.2 µg and 1 µg, respectively); lane 3 and 4, DNA of MFY30 strain (0.2 µg and 1 µg, respectively); lane 5 and 6, DNA of MFY32 strain (0.2 µg and 1 µg, respectively); lane 7, *H. wingei* chromosomes (Biorad, USA); lane 8 and 9, DNA of R2f strain (0.2 µg and 1 µg, respectively); lane 10 and 11, DNA of KT2440 strain (0.2 µg and 1 µg, respectively).
(PDF)

Figure S3 **Identification of tandem repeat.** In order to identified the tandem repeats, genomic DNAs were amplified by PCR using 28r (CTG AGC CAG GAT CAA ACT CT) and 1521f (TGC GGC TGG ATC ACC TCC TT) which correspond to the inverse-complementary of the universal 16S rRNA primers 8f and 1541r (Figure S1B). To test this protocol, we carried out an additional amplification in the same conditions except that the 1521f primer was replaced by the KT20kb primer (TAG GCA ACC CGT TCG ATA CT), a primer designed especially to obtain a PCR product of about 20 kb from the KT2440 strain (in combination with the 28r primer). (**A**) Test of the Crimson LongAmp *Taq* DNA polymerase (primers 28r / KT20kb): lane 1, negative control; lane 2, size ladder (Smart Ladder, Eurogenetec, Belgium); lane 3, KT2440 strain; and lane 4, Lambda genome digested by *Hind*III. (**B**) Search for tandem repeat (primers 28r / 1521f): lane 1, MF0 strain; lane 2, MFY30 strain; lane 3, MFY32 strain; lane 4, R2f strain; lane 5, KT2440 strain; lane 6, negative control; and lane 7, size ladder (Smart Ladder, Eurogenetec, Belgium).
(PDF)

Figure S4 **Secondary structure of the two different alleles of 16S rRNA in the MFY30 strain.** The helix (position 450 to 482 in the *E. coli* numbering system) contains the variable V3 motif. Secondary structure was determined using the mfold v2.3 software (Zuker, 2003) with default parameters except for the temperature (30°C instead of 37°C). Variable nucleotides are in bold print.
(PDF)

Figure S5 **Expression of the two different alleles of 16S rRNA in the MFY30 strain.** Expression of the two different copies (alleles a and b) was evaluated with sets of primers MFY30f (TCA GAT GAG CCT AGG TCG) / MFY30ar (TGC AGA GTA TTA ATC TAC AAC C) and MFY30f / MFY30br (ACT AAC GTA TTA GGT TAA TGC) led to the amplification of a fragment of the alleles a and b, respectively. Lane 1–5, RT-PCR with a set of primers specific to allele a (MFY30f / MFY30ar); lane 7–11, RT-PCR with a set of primers specific to allele b (MFY30f / MFY30br); lane 6, size ladder (Smart Ladder, Eurogenetec, Belgium); and lane 12, negative controls of both RT-PCR were pooled. Lane 1 and 7, clone MFY30b; lane 2 and 8, clone MFY30a; lane 3 and 9, extracted ARN; lane 4 and 10, cDNA; lane 5 and 11, genomic DNA.
(PDF)

Figure S6 **Phylogenetic relationships among the full-length 16S ribosomal RNA genes.** Only the genome-sequenced strains of *Pseudomonas* (highlighted by diamonds) and 59 *Pseudomonas* type strains (only the full-length 16S rRNA sequences from the Figure 1) were included. Compared to the Figure 1, the sequences from our studied strains and from twenty type strains were excluded. The colour of the symbol corresponds the 16S rRNA copy number in the given strain: white for a strain with 4 copies, light grey for a strain with 5 copies, dark grey for a strain with 6 copies, and black for a strain with 7 copies. The different alleles in the genome-sequenced strains are identified by a letter after the name of the strain, followed by the copy number of this corresponding allele. Bold print and arrows mark the three sequences from the aeruginosa r-cluster that are move to the fluorescens r-cluster when the V1 region is included in the Neighbour-Joining analysis. The unrooted dendrogram was estimated using the Neighbour-Joining algorithm from evolutionary distances computed according to the Jukes and Cantor correction. Numbers on tree branches report bootstrap results from Neighbour-Joining (above branch, 1000 replicates).
(PDF)

Figure S7 **Phylogenetic relationships among 1803 16S RNA sequences available in database.** The phylogenetic tree was built from 1803 full-length sequences available in database, excluding the V1 hypervariable region. The dataset included the 16S RNA gene of 79 *Pseudomonas* type strains that are highlighted according to their r-cluster: dark blue, green or red filled circles for the aeruginosa, fluorescens or putida r-cluster, respectively (deduced from Bodilis et al, 2004, 2011). The unrooted dendrogram was estimated using the Neighbour-Joining algorithm from evolutionary distances computed according to the Jukes and Cantor correction. The colour of the symbol corresponds to the proposed positions of the sequences in the aeruginosa (blue circle), fluorescens (green circle) and putida (red circle) r-clusters. The positions of the 1803 sequences in the different r-clusters were the same when a Maximum Likelihood analysis was carried out, expect for three sequences (highlighted by yellow triangles). This phylogenetic tree is the same as those of the Figures 4, 5 and S8.
(PDF)

Figure S8 **Phylogenetic occurrence of the major V6 hypervariable motif.** The phylogenetic tree was built from 1803 full-length 16S rRNA sequences of *Pseudomonas* available in the databases, after excluding the V1 region (see Fig. S7). The sequences highlighted by red circles have the only V6 motif found in at least two r-clusters (see Table 4).
(PDF)
