# Supplementary material for: Variable Copy Number, Intra-Genomic Heterogeneities and Lateral Transfers of the 16S rRNA Gene in Pseudomonas
Source: PLoS One. 2012 Apr 24;7(4):e35647. doi: 10.1371/journal.pone.0035647 (PMC3335818; doi:10.1371/journal.pone.0035647)

Figure S1. Southern blot analysis of rRNA operons in four *Pseudomonas* sp. strains. (**A**) Genomic DNAs were cleaved using restriction enzymes and hybridized with the 23S 3'end probe as described in the text. Lane 1, MF0 strain cleaved with *Pst*I + *Mlu*I ; lane 2, MFY30 cleaved with *Apa*I ; lane 3, MFY32 cleaved with *Pst*I ; lane 4, R2f cleaved with *Cla*I. (**B**) Schematic positions of rRNA probes.

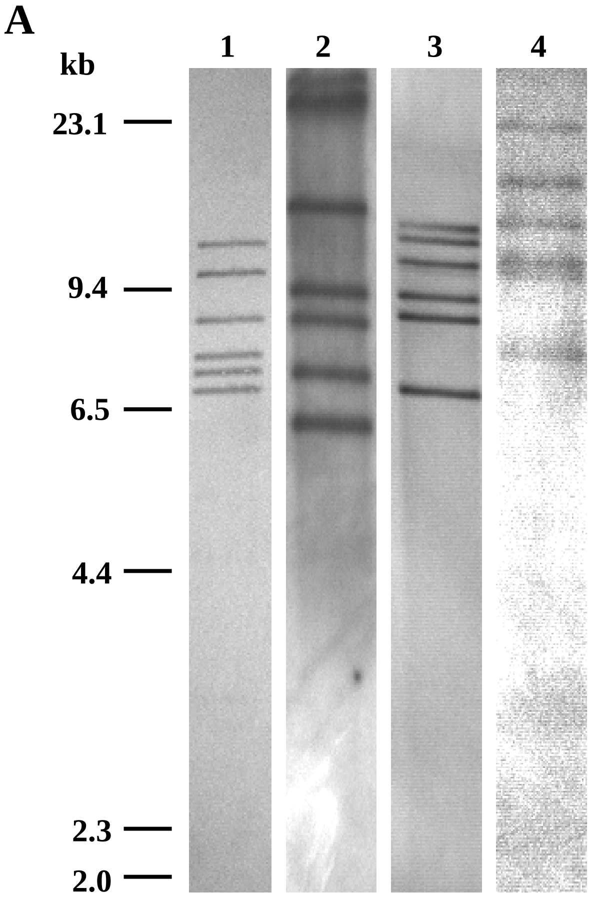

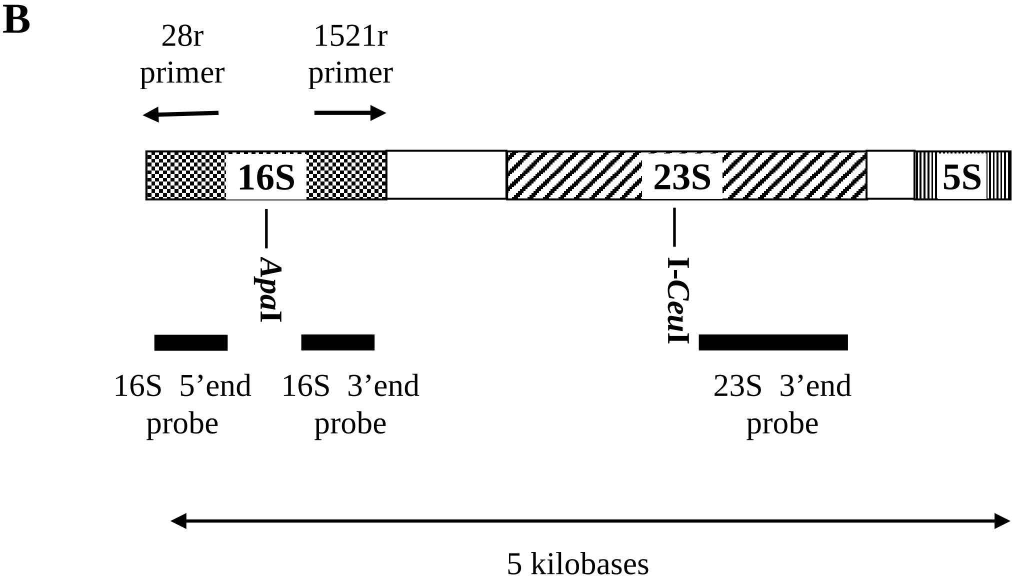

Supplement: Figure S1 — Southern blot analysis of rRNA operons in four Pseudomonas sp. strains. (A) Genomic DNAs were cleaved using restriction enzymes and hybridized with the 23S 3′end probe as described in the text. Lane 1, MF0 strain cleaved with PstI+MluI; lane 2, MFY30 cleaved with ApaI; lane 3, MFY32 cleaved with PstI; lane 4, R2f cleaved with ClaI. (B) Schematic positions of rRNA probes. (PDF) [file pone.0035647.s001.pdf]
