# Supplementary material for: Variable Copy Number, Intra-Genomic Heterogeneities and Lateral Transfers of the 16S rRNA Gene in Pseudomonas
Source: PLoS One. 2012 Apr 24;7(4):e35647. doi: 10.1371/journal.pone.0035647 (PMC3335818; doi:10.1371/journal.pone.0035647)

Figure S2. PFGE analysis of rRNA operons in the four *Pseudomonas* sp. strains. (**A**) Running conditions: 100 V for 40 h; pulse times, 80 s for 18 h, 100 s for 10 h, and 120 s for 12 h. Lane 1 and 2, DNA of MFY32 strain (0.2 µg and 1 µg, respectively); lane 3 and 4, DNA of R2f strain (0.2 µg and 1 µg, respectively); lane 5 and 6, DNA of MF0 strain (0.2 µg and 1 µg, respectively); lane 7, lambda DNA concatemers (Biorad, USA); Lane 8 and 9, DNA of MFY30 strain (0.2 µg and 1 µg, respectively); lane 10 and 11, DNA of KT2440 strain (0.2 µg and 1 µg, respectively). (**B**) Running conditions: 80 V for 70 h; linearly ramped pulse from 400 s to 800 s. Lane 1 and 2, DNA of MF0 strain (0.2 µg and 1 µg, respectively); lane 3 and 4, DNA of MFY30 strain (0.2 µg and 1 µg, respectively); lane 5 and 6, DNA of MFY32 strain (0.2 µg and 1 µg, respectively); lane 7, *H. wingei* chromosomes (Biorad, USA); lane 8 and 9, DNA of R2f strain (0.2 µg and 1 µg, respectively); lane 10 and 11, DNA of KT2440 strain (0.2 µg and 1 µg, respectively).

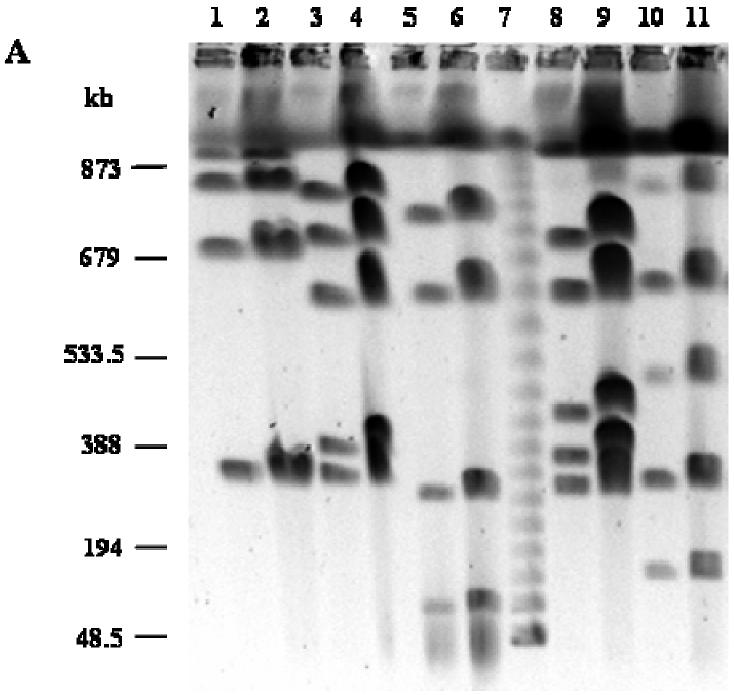

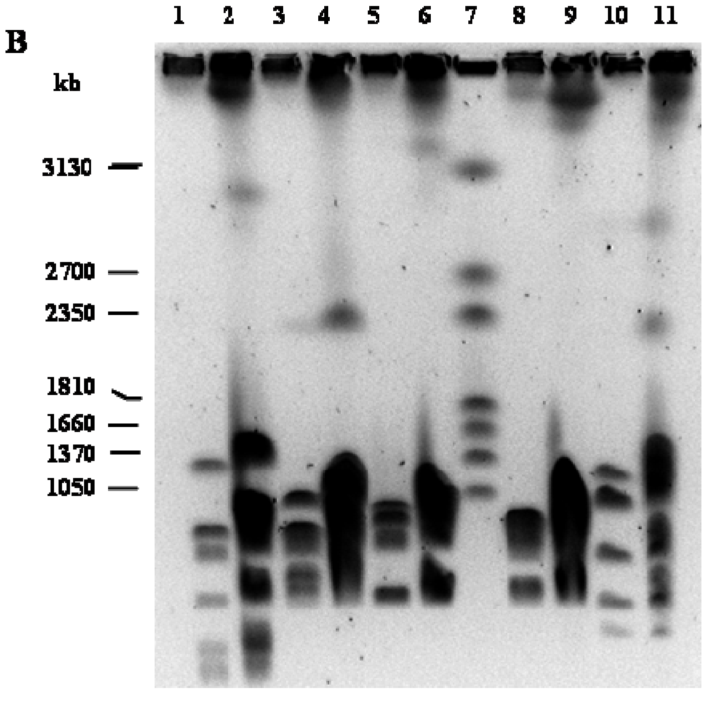

Supplement: Figure S2 — PFGE analysis of rRNA operons in the four Pseudomonas sp. strains. (A) Running conditions: 100 V for 40 h; pulse times, 80 s for 18 h, 100 s for 10 h, and 120 s for 12 h. Lane 1 and 2, DNA of MFY32 strain (0.2 µg and 1 µg, respectively); lane 3 and 4, DNA of R2f strain (0.2 µg and 1 µg, respectively); lane 5 and 6, DNA of MF0 strain (0.2 µg and 1 µg, respectively); lane 7, lambda DNA concatemers (Biorad, USA); Lane 8 and 9, DNA of MFY30 strain (0.2 µg and 1 µg, respectively); lane 10 and 11, DNA of KT2440 strain (0.2 µg and 1 µg, respectively). (B) Running conditions: 80 V for 70 h; linearly ramped pulse from 400 s to 800 s. Lane 1 and 2, DNA of MF0 strain (0.2 µg and 1 µg, respectively); lane 3 and 4, DNA of MFY30 strain (0.2 µg and 1 µg, respectively); lane 5 and 6, DNA of MFY32 strain (0.2 µg and 1 µg, respectively); lane 7, H. wingei chromosomes (Biorad, USA); lane 8 and 9, DNA of R2f strain (0.2 µg and 1 µg, respectively); lane 10 and 11, DNA of KT2440 strain (0.2 µg and 1 µg, respectively). (PDF) [file pone.0035647.s002.pdf]
