# Supplementary material for: Variable Copy Number, Intra-Genomic Heterogeneities and Lateral Transfers of the 16S rRNA Gene in Pseudomonas
Source: PLoS One. 2012 Apr 24;7(4):e35647. doi: 10.1371/journal.pone.0035647 (PMC3335818; doi:10.1371/journal.pone.0035647)

Figure S3. Identification of tandem repeat. In order to identified the tandem repeats, genomic DNAs were amplified by PCR using 28r (CTG AGC CAG GAT CAA ACT CT) and 1521f (TGC GGC TGG ATC ACC TCC TT) which correspond to the inverse-complementary of the universal 16S rRNA primers 8f and 1541r (Figure S1B). To test this protocol, we carried out an additional amplification in the same conditions except that the 1521f primer was replaced by the KT20kb primer (TAG GCA ACC CGT TCG ATA CT), a primer designed especially to obtain a PCR product of about 20 kb from the KT2440 strain (in combination with the 28r primer). (**A**) Test of the Crimson LongAmp *Taq* DNA polymerase (primers 28r / KT20kb): lane 1, negative control; lane 2, size ladder (Smart Ladder, Eurogenetec, Belgium); lane 3, KT2440 strain; and lane 4, Lambda genome digested by *Hind*III. (**B**) Search for tandem repeat (primers 28r / 1521f): lane 1, MF0 strain; lane 2, MFY30 strain; lane 3, MFY32 strain; lane 4, R2f strain; lane 5, KT2440 strain; lane 6, negative control; and lane 7, size ladder (Smart Ladder, Eurogenetec, Belgium).

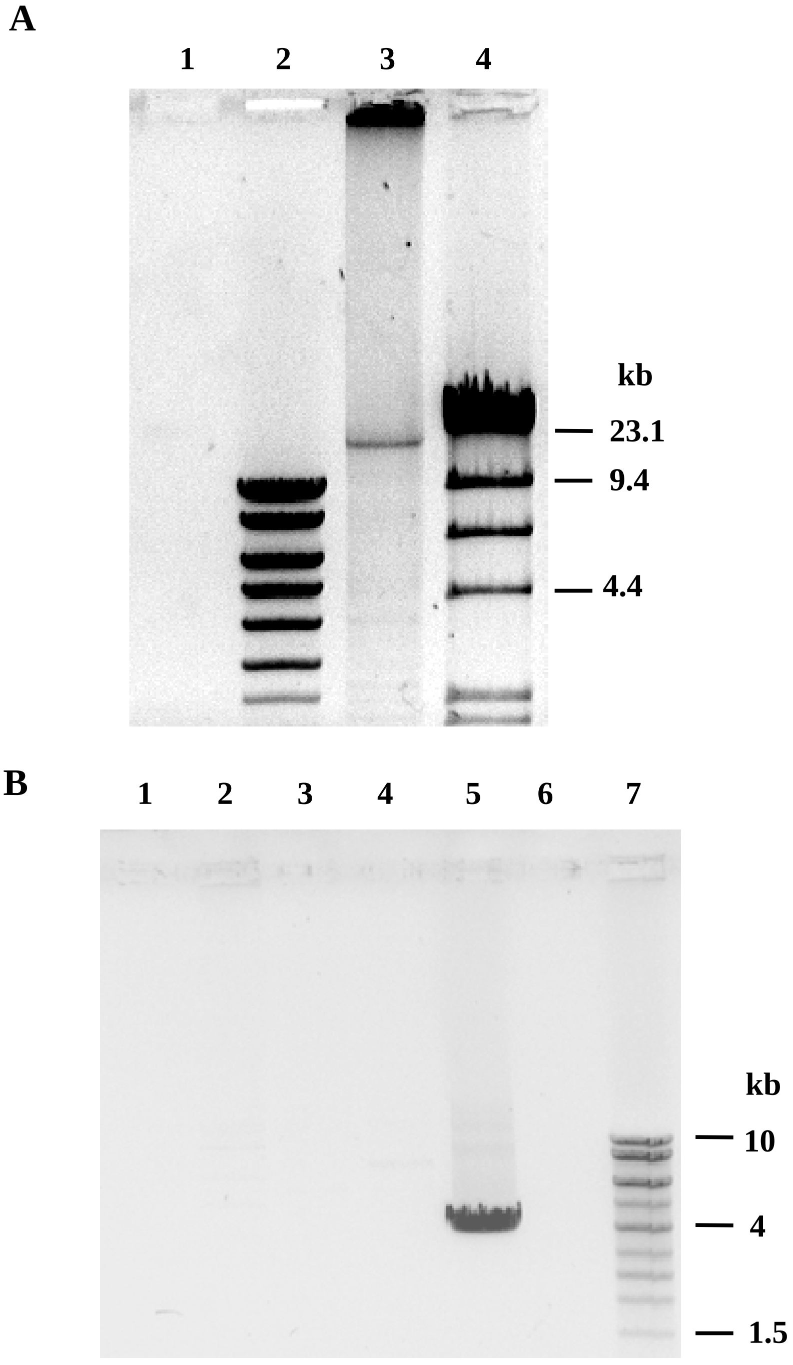

Supplement: Figure S3 — Identification of tandem repeat. In order to identified the tandem repeats, genomic DNAs were amplified by PCR using 28r (CTG AGC CAG GAT CAA ACT CT) and 1521f (TGC GGC TGG ATC ACC TCC TT) which correspond to the inverse-complementary of the universal 16S rRNA primers 8f and 1541r (Figure S1B). To test this protocol, we carried out an additional amplification in the same conditions except that the 1521f primer was replaced by the KT20kb primer (TAG GCA ACC CGT TCG ATA CT), a primer designed especially to obtain a PCR product of about 20 kb from the KT2440 strain (in combination with the 28r primer). (A) Test of the Crimson LongAmp Taq DNA polymerase (primers 28r / KT20kb): lane 1, negative control; lane 2, size ladder (Smart Ladder, Eurogenetec, Belgium); lane 3, KT2440 strain; and lane 4, Lambda genome digested by HindIII. (B) Search for tandem repeat (primers 28r / 1521f): lane 1, MF0 strain; lane 2, MFY30 strain; lane 3, MFY32 strain; lane 4, R2f strain; lane 5, KT2440 strain; lane 6, negative control; and lane 7, size ladder (Smart Ladder, Eurogenetec, Belgium). (PDF) [file pone.0035647.s003.pdf]
