# Supplementary material for: Variable Copy Number, Intra-Genomic Heterogeneities and Lateral Transfers of the 16S rRNA Gene in Pseudomonas
Source: PLoS One. 2012 Apr 24;7(4):e35647. doi: 10.1371/journal.pone.0035647 (PMC3335818; doi:10.1371/journal.pone.0035647)
Supplement: Figure S4 — Secondary structure of the two different alleles of 16S rRNA in the MFY30 strain. The helix (position 450 to 482 in the E. coli numbering system) contains the variable V3 motif. Secondary structure was determined using the mfold v2.3 software (Zuker, 2003) with default parameters except for the temperature (30°C instead of 37°C). Variable nucleotides are in bold print. (PDF) [file pone.0035647.s004.pdf]

Figure S4. Secondary structure of the two different alleles of 16S rRNA in the MFY30 strain. The helix (position 450 to 482 in the *E. coli* numbering system) contains the variable V3 motif. Secondary structure was determined using the mfold v2.3 software (Zuker, 2003) with default parameters except for the temperature (30°C instead of 37°C).Variable nucleotides are in bold print.

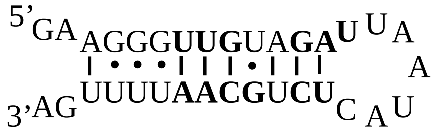

Allele a

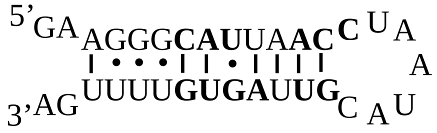

Allele b
