# Supplementary material for: Variable Copy Number, Intra-Genomic Heterogeneities and Lateral Transfers of the 16S rRNA Gene in Pseudomonas
Source: PLoS One. 2012 Apr 24;7(4):e35647. doi: 10.1371/journal.pone.0035647 (PMC3335818; doi:10.1371/journal.pone.0035647)

Figure S5. Expression of the two different alleles of 16S rRNA in the MFY30 strain. Expression of the two different copies (alleles a and b) was evaluated with sets of primers MFY30f (TCA GAT GAG CCT AGG TCG) / MFY30ar (TGC AGA GTA TTA ATC TAC AAC C) and MFY30f / MFY30br (ACT AAC GTA TTA GGT TAA TGC) led to the amplification of a fragment of the alleles a and b, respectively. Lane 1-5, RT-PCR with a set of primers specific to allele a (MFY30f / MFY30ar); lane 7-11, RT-PCR with a set of primers specific to allele b (MFY30f / MFY30br); lane 6, size ladder (Smart Ladder, Eurogenetec, Belgium); and lane 12, negative controls of both RT-PCR were pooled. Lane 1 and 7, clone MFY30b; lane 2 and 8, clone MFY30a; lane 3 and 9, extracted ARN; lane 4 and 10, cDNA; lane 5 and 11, genomic DNA.

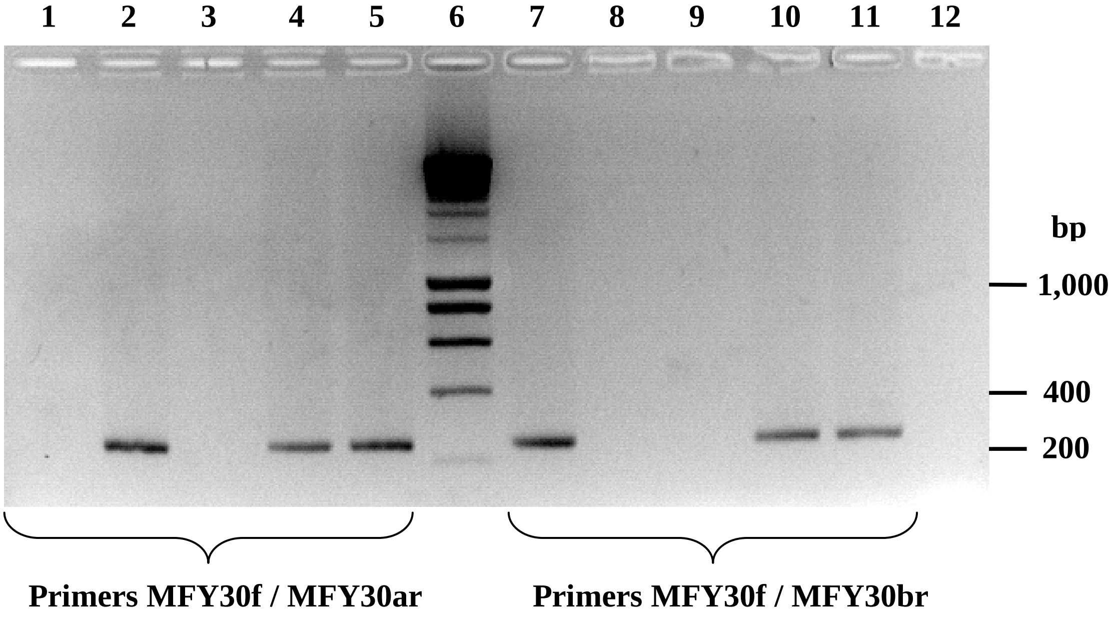

Supplement: Figure S5 — Expression of the two different alleles of 16S rRNA in the MFY30 strain. Expression of the two different copies (alleles a and b) was evaluated with sets of primers MFY30f (TCA GAT GAG CCT AGG TCG) / MFY30ar (TGC AGA GTA TTA ATC TAC AAC C) and MFY30f / MFY30br (ACT AAC GTA TTA GGT TAA TGC) led to the amplification of a fragment of the alleles a and b, respectively. Lane 1–5, RT-PCR with a set of primers specific to allele a (MFY30f / MFY30ar); lane 7–11, RT-PCR with a set of primers specific to allele b (MFY30f / MFY30br); lane 6, size ladder (Smart Ladder, Eurogenetec, Belgium); and lane 12, negative controls of both RT-PCR were pooled. Lane 1 and 7, clone MFY30b; lane 2 and 8, clone MFY30a; lane 3 and 9, extracted ARN; lane 4 and 10, cDNA; lane 5 and 11, genomic DNA. (PDF) [file pone.0035647.s005.pdf]
