# Supplementary figures and images for: Variable Copy Number, Intra-Genomic Heterogeneities and Lateral Transfers of the 16S rRNA Gene in Pseudomonas
Source: PLoS One. 2012 Apr 24;7(4):e35647. doi: 10.1371/journal.pone.0035647 (PMC3335818; doi:10.1371/journal.pone.0035647)

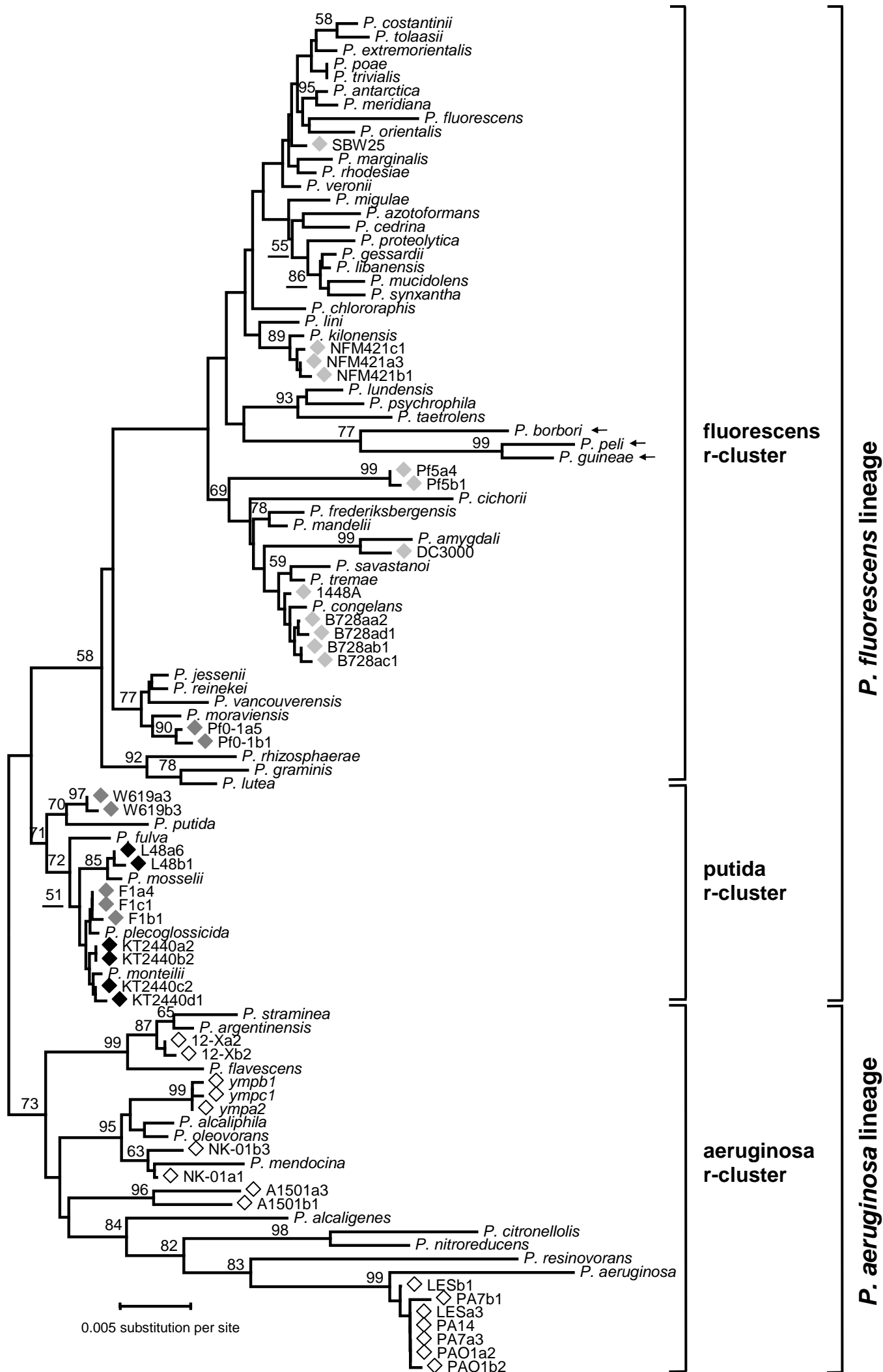

Figure S6. Phylogenetic relationships among the full-length 16S ribosomal RNA genes.

Supplement: Figure S6 — Phylogenetic relationships among the full-length 16S ribosomal RNA genes. Only the genome-sequenced strains of Pseudomonas (highlighted by diamonds) and 59 Pseudomonas type strains (only the full-length 16S rRNA sequences from the Figure 1) were included. Compared to the Figure 1, the sequences from our studied strains and from twenty type strains were excluded. The colour of the symbol corresponds the 16S rRNA copy number in the given strain: white for a strain with 4 copies, light grey for a strain with 5 copies, dark grey for a strain with 6 copies, and black for a strain with 7 copies. The different alleles in the genome-sequenced strains are identified by a letter after the name of the strain, followed by the copy number of this corresponding allele. Bold print and arrows mark the three sequences from the aeruginosa r-cluster that are move to the fluorescens r-cluster when the V1 region is included in the Neighbour-Joining analysis. The unrooted dendrogram was estimated using the Neighbour-Joining algorithm from evolutionary distances computed according to the Jukes and Cantor correction. Numbers on tree branches report bootstrap results from Neighbour-Joining (above branch, 1000 replicates). (PDF) [file pone.0035647.s006.pdf]
