# Supplementary material for: Variable Copy Number, Intra-Genomic Heterogeneities and Lateral Transfers of the 16S rRNA Gene in Pseudomonas
Source: PLoS One. 2012 Apr 24;7(4):e35647. doi: 10.1371/journal.pone.0035647 (PMC3335818; doi:10.1371/journal.pone.0035647)

Figure S7. Phylogenetic relationships among 1803 16S RNA sequences available in database. The phylogenetic tree was built from 1803 full-length sequences available in database, excluding the V1 hypervariable region. The dataset included the 16S RNA gene of 79 *Pseudomonas* type strains that are highlighted according to their r-cluster: dark blue, green or red filled circles for the aeruginosa, fluorescens or putida r-cluster, respectively (deduced from Bodilis et al, 2004, 2011). The unrooted dendrogram was estimated using the Neighbour-Joining algorithm from evolutionary distances computed according to the Jukes and Cantor correction. The colour of the symbol corresponds to the proposed positions of the sequences in the aeruginosa (blue circle), fluorescens (green circle) and putida (red circle) r-clusters. The positions of the 1803 sequences in the different r-clusters were the same when a Maximum Likelihood analysis was carried out, expect for three sequences (noted by yellow triangles). This phylogenetic tree is the same as those of the Figures 4, 5 and S8.

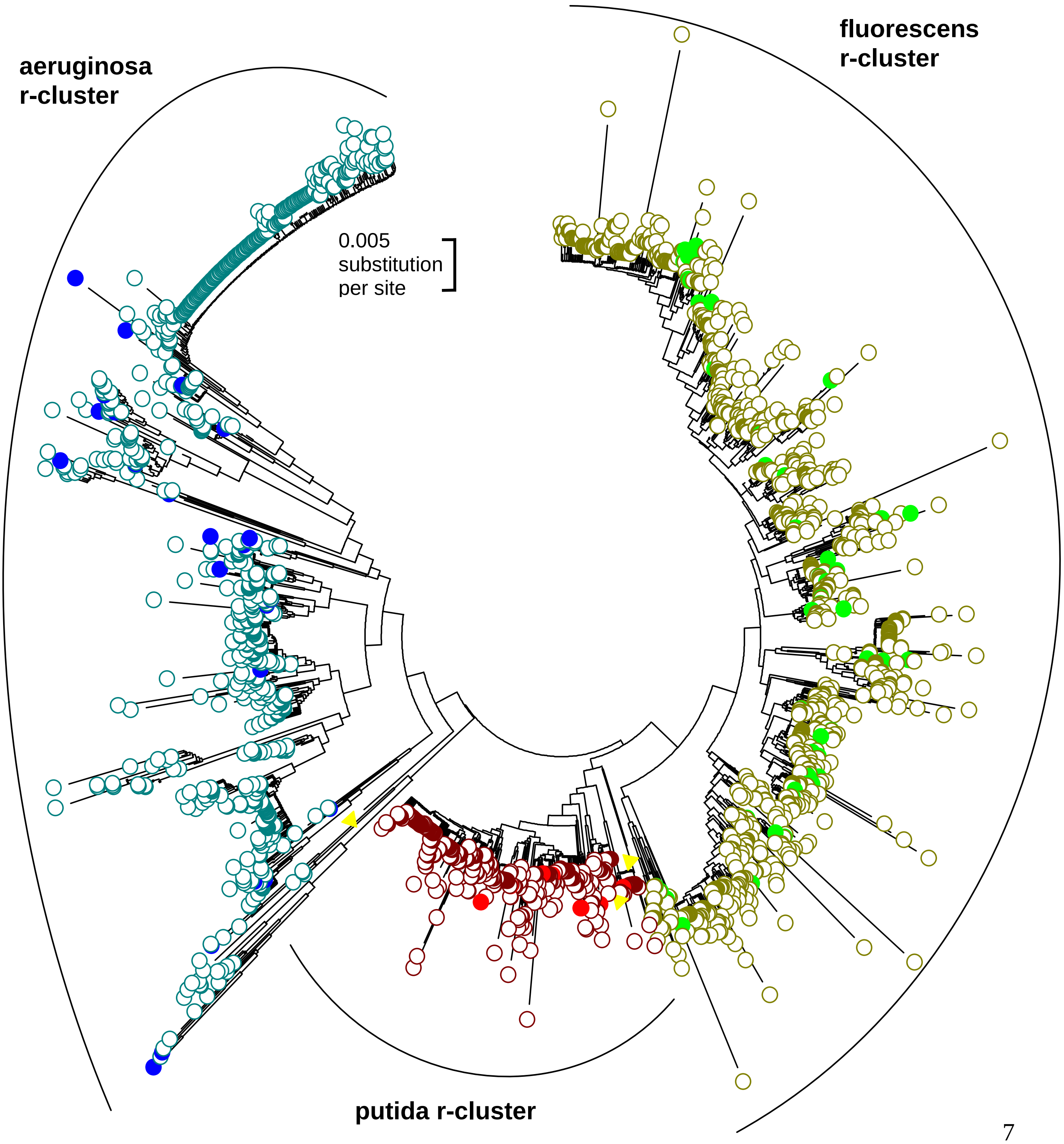

Supplement: Figure S7 — Phylogenetic relationships among 1803 16S RNA sequences available in database. The phylogenetic tree was built from 1803 full-length sequences available in database, excluding the V1 hypervariable region. The dataset included the 16S RNA gene of 79 Pseudomonas type strains that are highlighted according to their r-cluster: dark blue, green or red filled circles for the aeruginosa, fluorescens or putida r-cluster, respectively (deduced from Bodilis et al, 2004, 2011). The unrooted dendrogram was estimated using the Neighbour-Joining algorithm from evolutionary distances computed according to the Jukes and Cantor correction. The colour of the symbol corresponds to the proposed positions of the sequences in the aeruginosa (blue circle), fluorescens (green circle) and putida (red circle) r-clusters. The positions of the 1803 sequences in the different r-clusters were the same when a Maximum Likelihood analysis was carried out, expect for three sequences (highlighted by yellow triangles). This phylogenetic tree is the same as those of the Figures 4, 5 and S8. (PDF) [file pone.0035647.s007.pdf]
