# Supplementary material for: Variable Copy Number, Intra-Genomic Heterogeneities and Lateral Transfers of the 16S rRNA Gene in Pseudomonas
Source: PLoS One. 2012 Apr 24;7(4):e35647. doi: 10.1371/journal.pone.0035647 (PMC3335818; doi:10.1371/journal.pone.0035647)

Figure S8. Phylogenetic occurrence of the major V6 hypervariable motif. The phylogenetic tree was built from 1803 full-length 16S rRNA sequences of *Pseudomonas* available in the databases, after excluding the V1 region (see Fig. S7). The sequences noted by a red circle have the only V6 motif found in at least two r-clusters (see Table 4).

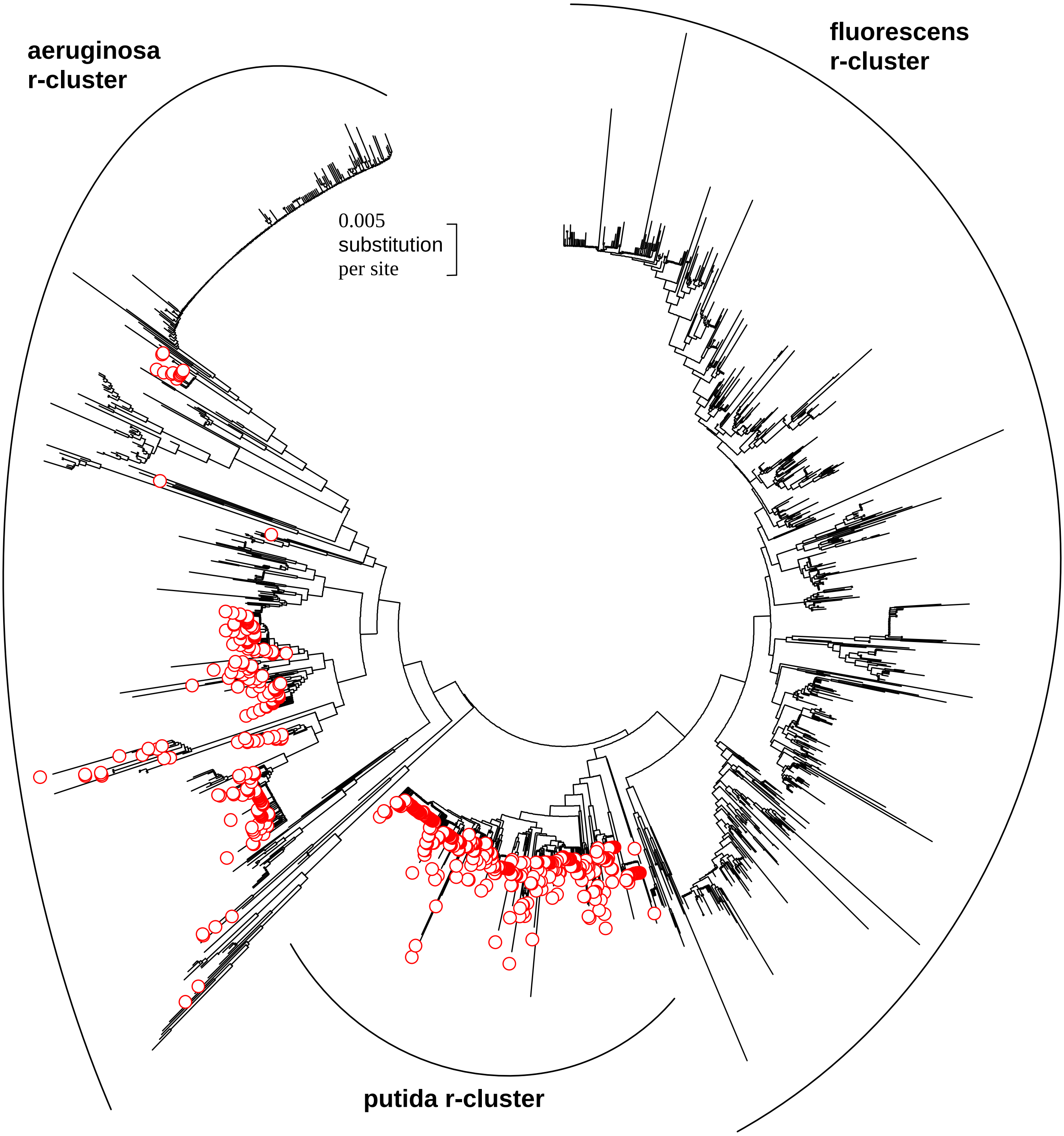

Supplement: Figure S8 — Phylogenetic occurrence of the major V6 hypervariable motif. The phylogenetic tree was built from 1803 full-length 16S rRNA sequences of Pseudomonas available in the databases, after excluding the V1 region (see Fig. S7). The sequences highlighted by red circles have the only V6 motif found in at least two r-clusters (see Table 4). (PDF) [file pone.0035647.s008.pdf]
